# A general mechanism for intracellular toxicity of metal-containing nanoparticles†
†Electronic supplementary information (ESI) available. See DOI: 10.1039/c4nr01234h



**DOI:** 10.1039/c4nr01234h

**Published:** 2014-05-20

**Authors:** Stefania Sabella, Randy P. Carney, Virgilio Brunetti, Maria Ada Malvindi, Noura Al-Juffali, Giuseppe Vecchio, Sam M. Janes, Osman M. Bakr, Roberto Cingolani, Francesco Stellacci, Pier Paolo Pompa

**Affiliations:** a Istituto Italiano di Tecnologia , Center for Bio-Molecular Nanotechnologies@UniLe , Via Barsanti , 73010 Arnesano (Lecce) , Italy . Email: pierpaolo.pompa@iit.it ; Fax: +39-0832-1816230 ; Tel: +39-0832-1816214; b Institute of Materials , École Polytechnique Fédérale de Lausanne (EPFL) , CH-1015 Lausanne , Switzerland . Email: francesco.stellacci@epfl.ch ; Fax: +41 21 6935270 ; Tel: +41 21 6937872; c Centre For Respiratory Research , Rayne Institute , University College London , 5 University Street , London WC1E 6JJ , UK; d Division of Physical Sciences and Engineering , Solar and Photovoltaics Engineering Center , King Abdullah University of Science and Technology (KAUST) , Thuwal 23955-6900 , Saudi Arabia; e Istituto Italiano di Tecnologia , Central Research Laboratories , Via Morego , 30-16136 Genova , Italy

## Abstract

The assessment of the risks exerted by nanoparticles is a key challenge for academic, industrial, and regulatory communities worldwide. Experimental evidence points towards significant toxicity for a range of nanoparticles both *in vitro* and *in vivo*. Worldwide efforts aim at uncovering the underlying mechanisms for this toxicity. Here, we show that the intracellular ion release elicited by the acidic conditions of the lysosomal cellular compartment – where particles are abundantly internalized – is responsible for the cascading events associated with nanoparticles-induced intracellular toxicity. We call this mechanism a “lysosome-enhanced Trojan horse effect” since, in the case of nanoparticles, the protective cellular machinery designed to degrade foreign objects is actually responsible for their toxicity. To test our hypothesis, we compare the toxicity of similar gold particles whose main difference is in the internalization pathways. We show that particles known to pass directly through cell membranes become more toxic when modified so as to be mostly internalized by endocytosis. Furthermore, using experiments with chelating and lysosomotropic agents, we found that the toxicity mechanism for different metal containing NPs (such as metallic, metal oxide, and semiconductor NPs) is mainly associated with the release of the corresponding toxic ions. Finally, we show that particles unable to release toxic ions (such as stably coated NPs, or diamond and silica NPs) are not harmful to intracellular environments.

## Abstract 2

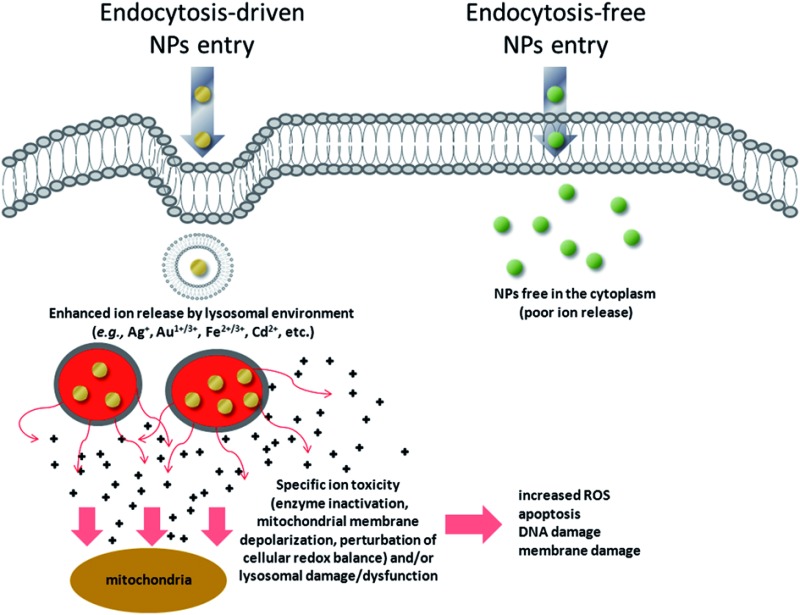
We demonstrate a general mechanism for the toxicity induced by metal-containing NPs, named “lysosome-enhanced Trojan horse effect”, which provides design rules to engineer safer NPs.

The growing use of nanotechnology in a wide range of industrial applications is raising concerns about the safety of nanoparticles (NPs). Recent outcomes of cross-disciplinary research suggest significant toxicity of NPs both in *in vitro* and *in vivo* systems.^1–3^ Hence, to prevent possible harmful effects, the research community, regulatory bodies, and all the stakeholders have been recently challenged to provide a reliable risk assessment of NPs.^4^ Large efforts are currently devoted to understanding the main issues in nanotoxicology accounting for the potential adverse effects of NPs to humans and to the environment. Several key findings have been made recently. Among these, it has been observed that NPs may trigger oxidative stress,^5,6^ inflammation,^7^ and indirect DNA damage^8^ in living systems, and that the size and shape of NPs may have a key role in determining the cellular damage.^2,9,10^ Moreover, it was demonstrated that the physico-chemical properties of NPs modulate their dynamic interaction with biomolecules and cellular organelles and, possibly, their toxicity.^5,11–13^ The formation of a biomolecular corona around the surface of NPs is a major element that defines their biological identity in biological fluids^14–16^ and their impact on biological functionalities.^17^ A protein corona may reduce cell uptake,^12,18^ induce protein unfolding triggering inflammation,^7^ or in some cases mitigate the cellular damage.^18–21^ It is noteworthy that some molecular mechanisms have recently emerged as new paradigms to explain NP injury, which involve lysosomal damage and autophagy.^20,22,23^ For instance, toxicity caused by the “proton sponge effect” has been demonstrated for cationic polymeric NPs accumulated in lysosomes.^22^ Interestingly, such an effect may be reduced by the presence of a protein corona around the NPs, which protects lysosomes from the bare surface of the NPs until protein degradation occurs.^20^ However, although much progress has been made in explaining NPs toxicity, a satisfactory integration of the various experimental observations as well as a general description of the mechanism underlying NPs toxicity is extremely challenging, due to the wide variety of available data, complex interactions involved, broad range of engineered NPs, and technical issues that may affect *in vitro* tests.^12,24,25^

In this work, we propose a general explanation for the toxicity of metal-containing NPs that could account for a large number of the reported observations; to the best of our knowledge, this is the first effort in this direction. We show that for a wide class of NPs (such as metallic, metal oxide, and semiconductor NPs) the acidic environment of the lysosomes triggers the release of relatively toxic ions (*e.g.*, Ag^+^, Cd^2+^, Fe^2+/3+^, Au^1+/3+^ ions) in the cell. We believe that these ions are the true mediators responsible for the observed intracellular toxicity profiles. Our model predicts that, once the NPs are abundantly taken-up in cells through active mechanisms of internalization^26,27^ (*i.e.*, the so-called Trojan horse effect),^23,28–30^ they can release intracellularly their cargo composed of toxic ions, as particle degradation is strongly promoted by the lysosomal environment. For this reason, we call this mechanism a “lysosome-enhanced Trojan horse effect” (LETH mechanism). This finding is quite general though counterintuitive since the cell's primary security process, engulfing and degrading the internalized foreign material *via* the acidic environment and proteases, instead results in overt nanoparticle toxicity. In particular, we show in a series of experiments that very similar AuNPs are significantly more toxic when entering cells *via* endocytosis as opposed to those mainly entering through energy independent mechanisms directly into the cytosol, and that for a large set of metal containing NPs their toxicity is mainly ascribed to their *in situ* degradation and intracellular release of toxic ions.

First, we investigated the time-dependent ion release of a variety of NPs, namely metallic (Au and Ag), magnetic (Fe_3_O_4_) and semiconductor (CdSe/ZnS) NPs. The synthesis and the physico-chemical characterizations of these NPs are reported in the Methods section in the ESI.† The ion leakage from the NPs was assessed by inductively coupled plasma atomic emission spectroscopy (ICP-AES) under two separate conditions, mimicking either the lysosomal environment (37 °C, pH 4.5)^31,32^ or the cellular cytoplasmic environment (37 °C, neutral pH) (see Methods section in the ESI† for details). As shown in Fig. 1, for all NPs tested, we observed significant ion release in the acidic conditions and no measurable release in neutral conditions (the NP behavior in neutral conditions was also tested in cell culture medium, DMEM, 10% FBS, pH 7.4, as a more relevant model of physiological conditions, and the same results were obtained). Such ion release was accompanied by obvious NP degradation, with consequent loss of NP morphology as well as of their fluorescence or magnetic properties (in the case of CdSe/ZnS or Fe_3_O_4_, respectively, see Fig. 1, bottom) (a more detailed analysis of NP degradation in an acidic environment is reported in the ESI, Fig. S1–S4†). The ion release profiles were specific to the NPs under investigation, depending on their core material, initial concentration, and specific coating. In any case, we found that the lysosomal environment is capable of promoting NP degradation/corrosion. We hypothesized that such ions, once released intracellularly, are likely to be the main factor in promoting the toxic effects of NPs (see below).

**Fig. 1:**
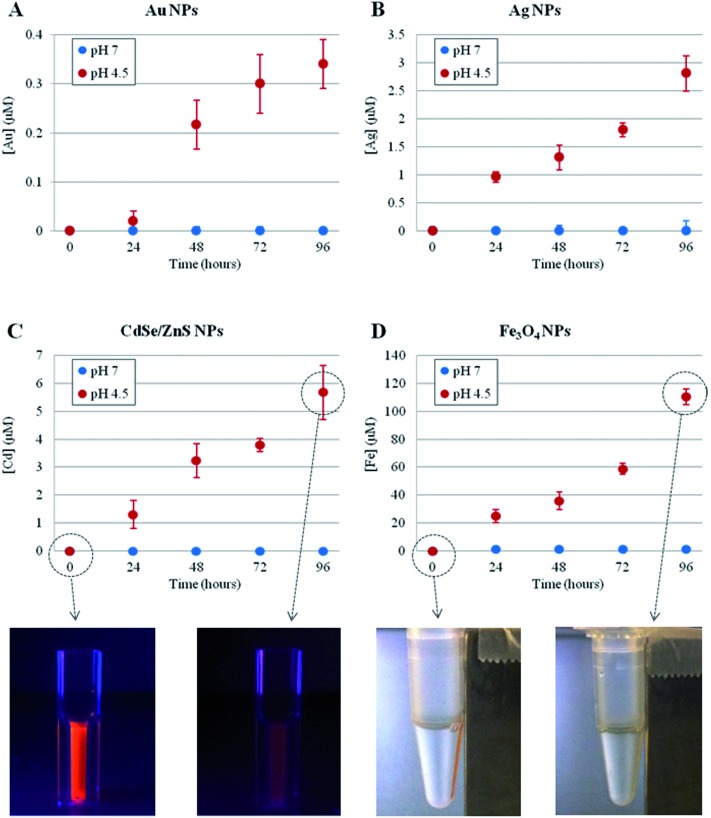
Time-dependent ion release, probed by ICP-AES, of different NPs at 37 °C, in neutral (blue symbols) or acidic (red symbols) conditions. (A) 4 nm AuNPs, 50 nM concentration (striped and unstructured AuNPs showed similar behavior, see also Fig. S5†); (B) 5 nm AgNPs, 17 nM concentration; (C) 6 nm CdSe/ZnS NPs, 20 nM concentration; (D) 10 nm Fe_3_O_4_ NPs, 40 nM concentration. The reported sizes of the NPs refer to their core structures (see Table S32 in the ESI†1). In (C) and (D) bottom, representative photographs of the respective NPs are also shown, at time 0 and after 96 h in acidic conditions, clearly revealing a significant loss of NPs' fluorescence (C) or magnetic (D) properties after the acidic treatment. Neutral and acidic conditions were obtained by dispersing the NPs in water (pH 7.0) or in citrate buffer (pH 4.5),^31,32^ respectively. Neutral conditions were also probed in cell culture medium (DMEM, 10% FBS, pH 7.4), obtaining the same results (*i.e.*, no detectable ion release). Data represent the average from 3 independent measurements (6 replicates for each experiment) and the error bars indicate the standard deviation.

To establish the role of lysosomal release of ions in the overall mechanism of NP toxicity, we performed several *in vitro* toxicity assays (*i.e.*, viability, ROS, and caspase assays) for cells incubated with two different types of AuNPs. These particles have identical physico-chemical properties (material, size, ligand shell density, zeta potential) but a slight difference in the ligand shell composition resulting in very different surface morphologies.^33–35^ The first type of particles were coated with a 2 : 1 molar mixture of 11-mercapto undecanesulfonic acid (MUS) and octanethiol (OT) and had a stripe-like morphology in their ligand shell, the second type of particles were coated with a 2 : 1 molar mixture of MUS and 2,7-dimethyl octanethiol (brOT) with a random distribution of molecules in the ligand shell. The first type of particles (“striped”) were shown to pass through cell membranes mostly *via* an energy independent mechanism; the latter ones (“unstructured”) could enter cells only by energy dependent processes, such as endocytosis.^36,37^ Striped and unstructured AuNPs were both tested in terms of the time-dependent ion release, and showed substantially the same behavior (see Fig. 1A and Fig. S5†). We evaluated the toxic effects of both AuNPs on monocytoid cells (U937), as well as on five other cell lines (see below). First, we verified that the cellular internalization of striped NPs is not affected by the inhibition of endocytotic processes, in contrast to unstructured particles that could not enter cells in these conditions (see Fig. S6†). Moreover, co-localization studies by confocal microscopy confirmed the classical lysosomal confinement of unstructured AuNPs, while striped AuNPs appear mainly distributed in the cytosol (Fig. S25 and 26†). Consistent with our toxicity model, U937 cell viability was not affected by striped AuNPs in the investigated concentration range, whilst unstructured particles caused a statistically significant time- and dose-dependent toxicity in the higher range of the investigated concentrations (Fig. 2A). Note that the striped and unstructured AuNPs were internalized in cells in similar quantities, as probed by ICP (Fig. S7†). The different toxicity behavior was further confirmed by the ROS assay, in which we observed an increase of the cellular ROS level upon treatment with unstructured NPs. Striped NPs induced ROS values close to the untreated control cells (Fig. 2B). Furthermore, as shown in Fig. 2C, we found that the unstructured AuNPs elicited a dose-dependent activation of caspase-3, whose occurrence converges on several events, including apoptosis *via* an intrinsic mitochondrial pathway.^38,39^ The same assays were also performed in other cell lines, namely human cervix carcinoma epithelial cells (HeLa), human breast adenocarcinoma epithelial cells (MCF7), human colon adenocarcinoma epithelial cells (Caco-2), human neuroblastoma cells (SH SY5Y), and human hepatoma cells (Huh-7), confirming the same NP behavior (Fig. S8–S12†). Such data corroborate the hypothesis that the induced NP toxicity is internalization mechanism-dependent, namely that endocytosed NPs entrapped in the lysosomes undergo enhanced corrosion and ion leakage, with consequent toxicity to cells.

**Fig. 2:**
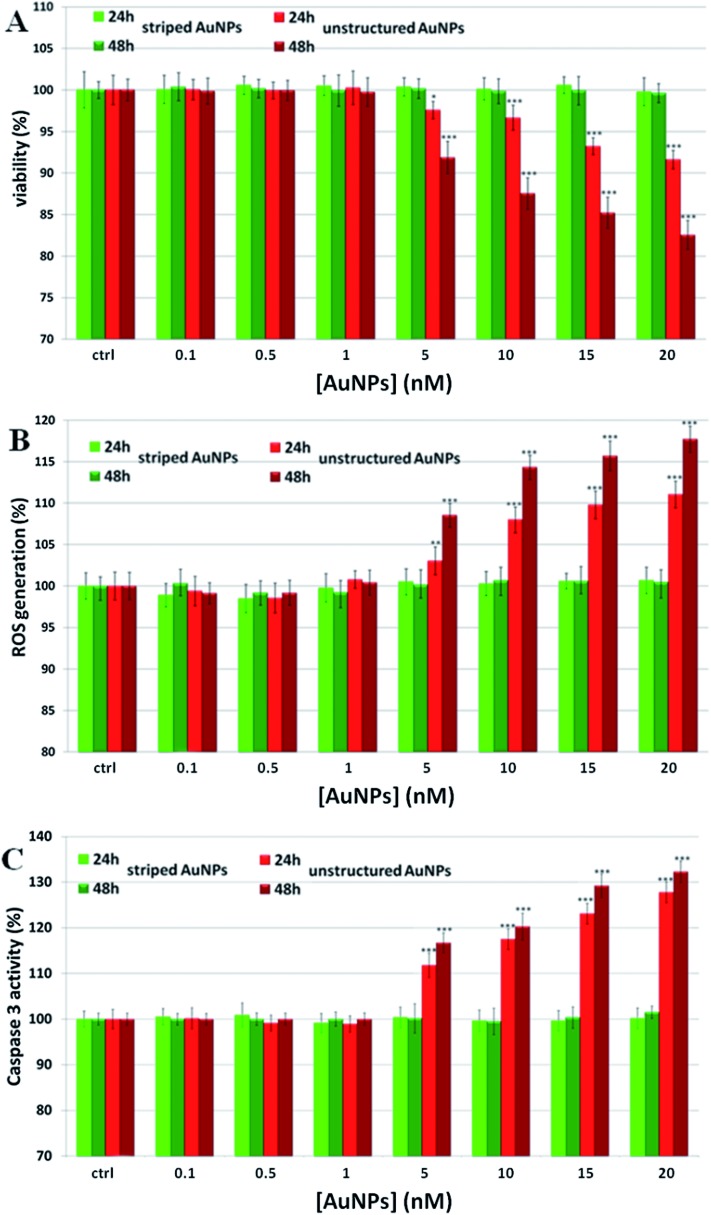
Toxicity assessment of striped and unstructured AuNPs in U937 cells. (A) WST-8 proliferation assay upon treatment with increasing amount of AuNPs. Ctrl represents the negative control; values are mean ± SD. Positive controls (not shown) were treated with 0.01% of TritonX100, displaying a strong viability decrease (*ca.* 80–90%) with respect to the untreated cells. (B) ROS quantification, *via* DCFH-DA assay, after cellular treatment with AuNPs; values are mean ± SD. Positive controls (not shown) were treated with a free radical generator (100 μM H_2_O_2_), exhibiting a ROS increase of *ca.* 190–220% with respect to the untreated control cells. (C) Evaluation of caspase 3 activity. Values are mean ± SD. Results were analyzed by Two-way ANOVA and values compared to the control by the Bonferroni post-hoc test. Differences between treated samples and controls (*n* = 8) were considered statistically significant for ***P < 0.001, **P < 0.01, *P < 0.05, and non-significant for *P* > 0.05.

To strengthen our comparison, we focused our studies on striped nanoparticles and modified them with transferrin (“Tstriped”) or apolipoprotein (“Astriped”). In both cases, the intent was to modify the particles in order to activate a receptor mediated endocytosis mechanism of uptake. We incubated cells with striped, Tstriped, and Astriped NPs. We point out that the particles are exactly the same, only differing in post-synthesis functionalization (the bioconjugates characterization is reported in the ESI†). Yet, only the Tstriped and Astriped particles showed significant toxicity (Fig. S13†).

The different toxicity induced by the striped and unstructured AuNPs was also verified *in vivo*, using the model system *Drosophila melanogaster*.^40^ Organisms were treated with AuNPs (dose: 0.36 μg g^–1^ per day, see Methods section in the ESI† for details) and their possible toxicity was evaluated by analyzing the lifespan of the different populations. Interestingly, we found that unstructured particles elicited a considerable lifespan reduction (the average lifespan, *τ*_50_, was *ca.* 47% lower than the control), while striped particles did not significantly affect the life cycle of the treated population (the small difference observed was not statistically significant), despite a similar bioaccumulation of the two AuNPs in the organisms (as probed by ICP measurements) (see Fig. S14†).

Overall, our model explains the toxicity of metal containing NPs in terms of intracellular release of the corresponding toxic ions. Hence, the toxicity mechanisms of NPs should follow the same molecular pathways triggered by the specific ions. In the case of AuNPs, we thus verified that the induced toxicity was mainly due to the same molecular mechanisms activated by gold ions (Au^1+/3+^). Intracellular gold ions are known to strongly inhibit the enzyme thioredoxin reductase (TrxR), leading to mitochondrial membrane depolarization and/or inactivation of mitochondrial enzymes^41^ (a more detailed description of the TrxR functionality and the impact of its inhibition on cell functions are reported in the ESI†). This, in turn, causes several toxic mainstream events, including alteration of cellular redox balance, increase of physiological ROS levels, and occurrence of apoptosis.^39^ We verified the inhibition of TrxR by AuNPs with two experiments. First, we analyzed the inhibition of the activity of extracted TrxR by the ions released by AuNPs in both neutral and acidic environments (namely in the conditions described in Fig. 1). We observed that the Au ions released in the acidic environment are capable of reducing the TrxR activity with respect to the control (*ca.* 35% of inhibition), while the negligible amount of ions generated in the neutral environment did not significantly affect the enzyme activity (Fig. 3A). This indicates that the released gold ions from the AuNPs are active against the enzyme. Furthermore, we were able to demonstrate that AuNPs can inhibit TrxR functionality directly in the cells. We analyzed the TrxR activity in HeLa cells after 48 h of incubation with the striped and unstructured AuNPs and also in this case, we observed that the unstructured AuNPs induced a clear reduction of the enzyme activity (Fig. 3B), at variance with the striped NPs showing a very weak enzyme inhibition. Such inhibition of TrxR functionality is consistent with the corresponding decrease in cell viability (see Fig. S15†). Analogous results were obtained by using another cell line, namely U937 (Fig. S16†). Furthermore, we found that a similar behavior, with comparable enzyme inhibition, occurs also by using citrate-capped AuNPs (Fig. S17†). The available evidence indicates that the TrxR system functionality is significantly compromised by the ion cargo released in the cells by the unstructured AuNPs (see also below), caused by their lysosomal confinement. The released ions, in combination with other factors, such as the formation of peroxide intermediates (that may occur at the NP surface and/or during NP degradation^5,42^) are likely to be the main causes eliciting direct or indirect mitochondrial damage and consequent cellular redox imbalance.

**Fig. 3:**
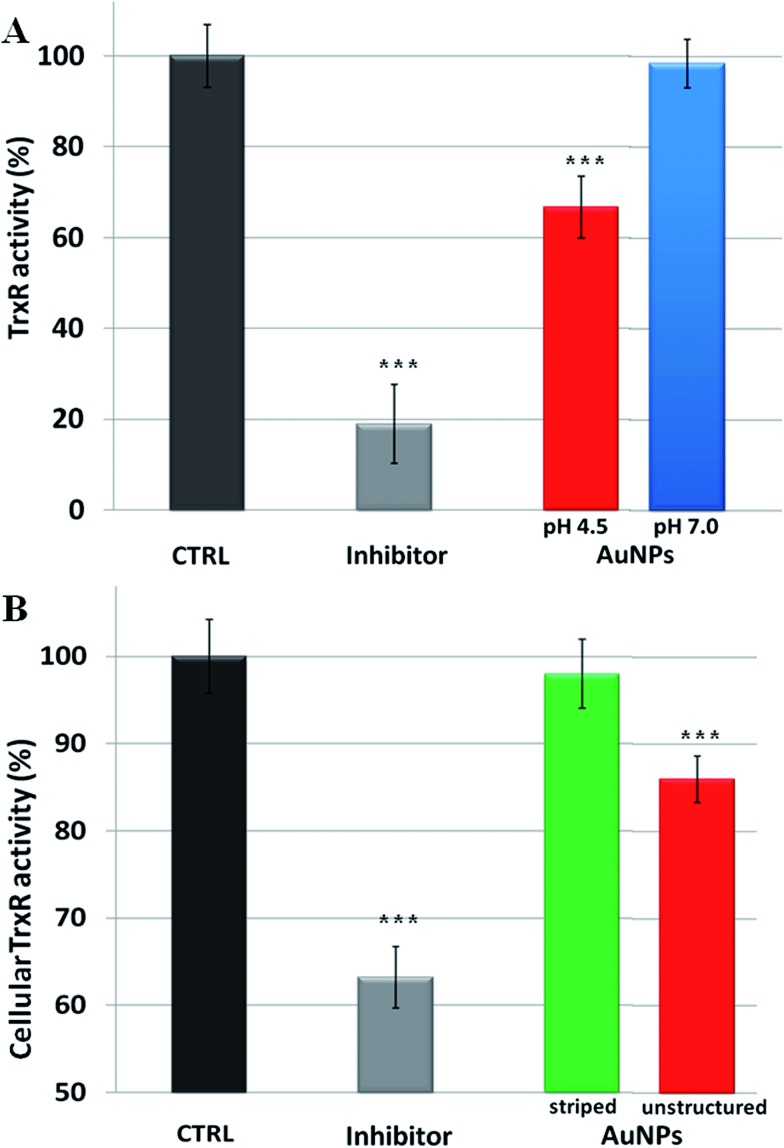
Inhibition of thioredoxin reductase (TrxR) by AuNPs. (A) Inhibition of TrxR activity by the gold ions released by AuNPs at different pH values (pH 4.5 or 7.0), according to the experiment reported in Fig. 1. The inhibitor drug auranofin was used as a positive control (see Methods section in the ESI†), while gold ion concentration at pH 4.5 was 20 nM (gold ion release was not detectable at pH 7.0). (B) Cellular TrxR activity in HeLa cells after 48 h treatment with striped and unstructured AuNPs (15 nM) and auranofin (1 μM) (see Methods section in the ESI† for experimental details). Results are mean ± SD and differences between treated samples and controls (*n* = 8) were considered statistically significant for ***P < 0.001.

The presence of different ion cargoes released intracellularly by the striped and unstructured AuNPs was quantified by measuring the amount of gold ions present in the cells upon treatment with AuNPs. As reported in Fig. 4, experimental data indicate that striped AuNPs do not release ions in the cell, whilst unstructured AuNPs, being entrapped in the lysosomes, are subjected to partial *in situ* degradation, leading to the release of their ion cargo (in agreement with Fig. 1). Such a cargo thus significantly contributes to NP toxicity to cells. Interestingly, these finding are also in agreement with the above data and discussion (*i.e.*, the presence of oxidative stress and apoptosis, Fig. 2B and C) and with recent observations reporting AuNPs-induced DNA damage and genotoxicity both *in vitro* and *in vivo*.^3,43,44^ However, the molecular mechanism involving TrxR blocking on mitochondria (reported here as a proof of principle to show toxicity induced by released gold ions) may not be the unique target of the gold ion cargo (see also below). In fact, we cannot exclude the possibility that the same ion cargo, released by the NPs, might also be responsible for other toxic mechanisms, such as lysosomal (and/or RER) damage, whose dysfunction may, in turn, activate other mainstream adverse events, in line with recent findings.^20,22,23^

**Fig. 4:**
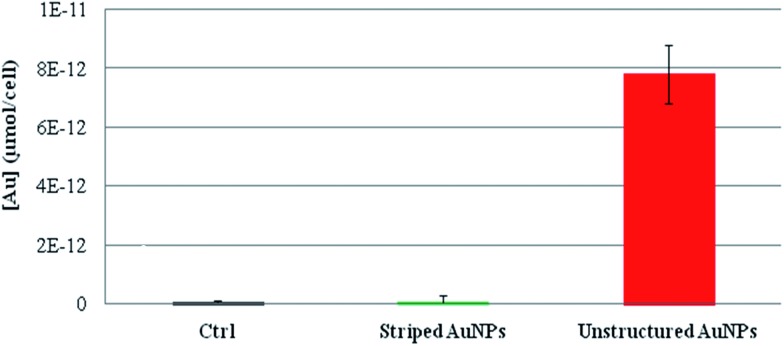
ICP-AES of intracellular gold ions released by striped and unstructured AuNPs upon internalization by HeLa cells. Cells were treated with striped and unstructured AuNPs (20 nM) for 48 hours. Upon incubation, cells were lysed and ultrafiltrated by Amicon Ultra-4 (experimental details are reported in the Methods section in the ESI†). The filtered solutions containing all the soluble ions were then analyzed by ICP-AES. The amount of gold ions is reported as μmol of filtered gold ions per cell. Results show that striped AuNPs do not release a measurable amount of ions, whereas unstructured AuNPs, being entrapped in the lysosomes, are subjected to partial acidic corrosion, leading to the release of their ion cargo.

We verified our model on several types of metal containing NPs (see Table S32† for a complete list of tested NPs and their relative physical–chemical characterization), observing that the toxicity can be mainly ascribed to that of the corresponding ions. First, as shown in Fig. 5, we probed the toxicity of the four types of NPs previously tested (*i.e.*, Au, Ag, Fe_3_O_4_, and CdSe/ZnS NPs) upon incubation with specific ion chelators.^45,46^ It is noteworthy that, for all the NPs tested, the presence of the chelating agents leads to a significant reduction of the NP toxicity. This indicates a major role of intracellularly released ions in eliciting NP toxicity. When the toxic ions are chelated, the toxic effects of the NPs are minimized. Note that, in the conditions employed in these experiments, the use of chelators did not affect the uptake efficiency of the tested NPs (see Fig. S18†), nor did it induce spurious off-target effects on cells (as shown in a “cross-toxicity test”, Fig. S19,† using specific and aspecific chelating molecules). Interestingly, and in agreement with previous studies,^22^ the ROS level and the cellular membrane integrity have also been found to be altered by NP treatments (Fig. S20†). Yet, these toxic outcomes are also drastically reduced by the presence of chelants, further demonstrating the central role of the *in situ* released ions in mediating cellular toxicity (see also below).

**Fig. 5:**
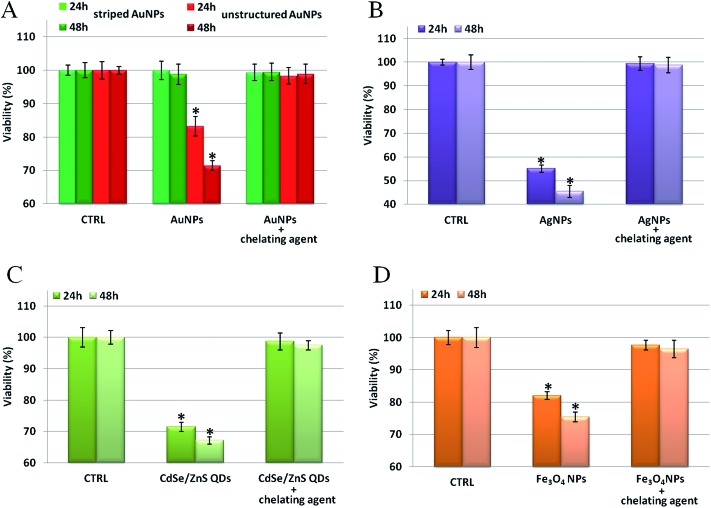
Toxicity assessment of different types of NPs in the absence/presence of specific ion chelators. WST-8 proliferation assays upon treatment with (A) striped and unstructured AuNPs (20 nM), (B) AgNPs (2 nM), and (C) CdSe/ZnS QDs (5 nM) in HeLa cells in the presence of 2,3-dithiopropanol (BAL); HeLa cells were pretreated for 30 min with/without 1 μM BAL and then exposed to the NPs for 24–48 h. (D) Proliferation assay upon treatment with 2.5 nM of Fe_3_O_4_ NPs. In this case, HeLa cells were pretreated for 30 min with/without 100 μM desferrioxamine (dfx) and then exposed to Fe_3_O_4_ NPs for 24–48 h. In all cases, the pretreatment with chelating agents suppresses, almost totally, the toxicity of the NPs. CTRL represents the negative control; values are mean ± SD. Differences between treated samples and controls (*n* = 8) were considered statistically significant for *P < 0.05 and non-significant for *P* > 0.05.

We also tested the general applicability of our model on other metal containing NPs, namely zinc oxide, aluminum oxide, platinum, and nickel NPs (see Table S32† for their physical–chemical characterization), confirming the above data. Notably, we observed that, while all these NPs strongly reduced cell viability, treatment with the chelating agents significantly reduced (and, in some cases, completely prevented) the NP induced cellular toxicity (Fig. S21†).

To further analyze the role of the acidic environment of lysosomes as a strong enhancer of NP corrosion and consequent release of toxic ions, once the NPs are actively internalized by cells, NP induced toxicity was assessed in the presence of lysosomotropic agents, which may prevent lysosomal acidification.^47^ In these experiments, cells were pretreated with two lysosomotropic agents (*i.e.*, chloroquine or ammonium chloride) enabling neutralization of lysosomal pH,^47^ and then exposed to unstructured AuNPs or Fe_3_O_4_ NPs. Interestingly, as shown in Fig. 6, we observed that the cellular toxicity of both NPs was strongly attenuated by the lysosomotropic agents, thanks to the neutralization of lysosomal acidity and consequent poor release of toxic ions in the cell. This means that the same NPs, at identical doses and cellular internalization, are significantly less toxic if the intracellular release of toxic ions is prevented.

**Fig. 6:**
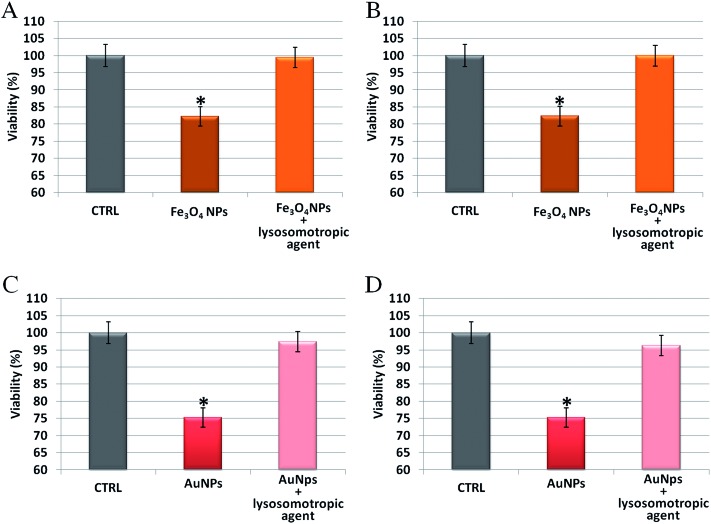
Toxicity assessment of unstructured AuNPs and Fe_3_O_4_ NPs in HeLa cells, in the presence/absence of two different lysosomotropic agents (chloroquine or ammonium chloride). (A and B) WST-8 proliferation assay upon treatment with Fe_3_O_4_ NPs (2.5 nM) and (C and D) unstructured AuNPs (20 nM). HeLa cells were pretreated for 30 min with/without 5 μM chloroquine (panels A and C) or 5 mM ammonium chloride (panels B and D) and then exposed to the NPs for 48 h. In all cases, NPs toxicity was strongly attenuated by the lysosomotropic agents that neutralize lysosomal acidity, preventing ion release and cytotoxicity. CTRL represents the negative control; values are mean ± SD. Differences between treated samples and controls (*n* = 8) were considered statistically significant for *P < 0.05 and non-significant for *P* > 0.05.

It should be noted that, in terms of cellular toxicity, the Trojan horse mechanism of NPs^23,28–30^ is mediated and enhanced by lysosomes (see also below). NPs are abundantly internalized by cells and then lysosomes elicit the intracellular release of ions with consequent toxicity to cells. If cells are exposed to the same or even higher doses of toxic ions, dispersed in the culture medium, no toxicity is typically detected. As an example, we tested the toxicity of gold salts up to a concentration *ca.* 3-fold higher than the maximum concentration used in all the cytotoxicity assays for AuNPs, and no toxicity was observed (Fig. S22†). This suggests that the ions cannot exert their toxicity because they lack the Trojan horse effect and cannot abundantly enter the cells.

We have, therefore, identified a key toxicity mechanism that we define as a “Lysosome-Enhanced Trojan Horse effect (LETH effect)” (Fig. 7). Such a mechanism may be valid for all those NPs that are actively and efficiently taken up by cells (*i.e.*, almost all NPs < 100 nm), whose acidic corrosion results in the generation of toxic ions. The LETH effect thus combines the abundant cellular internalization of the NPs *via* active processes with the consequent enhanced release of the relatively toxic ions (*e.g.*, Ag^+^, Cd^2+^, Fe^2+/3+^, Au^1+/3+^ ions) in the cytoplasm elicited by the acidic lysosomal environment. The induced cellular toxicity can be therefore mainly ascribed to that of the corresponding ions (as also recently suggested in Cd-based QDs and ZnO NPs^26,48,49^), with resulting oxidative stress and apoptosis. More generally, the significant amount of intracellularly leaked ions may exert ion-specific toxicity (*e.g.*, enzyme inactivation as demonstrated for gold ions) against cellular targets (*e.g.*, mitochondria, RER, *etc.*) and/or lysosomal damage/dysfunction, in line with recent findings with other types of NPs.^20,22,23^

**Fig. 7:**
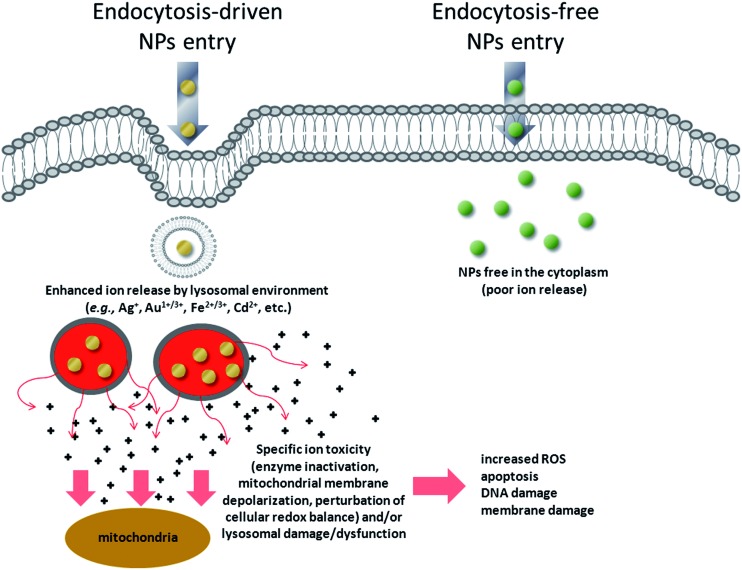
Schematic of the general toxicity mechanism induced by NPs when they enter cells by active internalization mechanisms, compared to endocytosis-free NPs. NPs that enter the cells by energy-dependent processes (mediated by clathrin, caveolin, lipid raft formations and others)^59^ are rapidly confined in vesicular structures, endosomes, and finally in lysosomes. The acidic lysosomal pH triggers a lysosome-enhanced Trojan horse effect (LETH effect) that combines the abundant cellular internalization of the NPs *via* active processes with the consequent enhanced release of the relatively toxic ions (*e.g.*, Ag^+^, Cd^2+^, Fe^2+/3+^, Au^1+/3+^ ions). The significant amount of intracellularly leaked ions may then exert ion-specific toxicity (*e.g.*, enzyme depletion/inactivation, protein denaturation, *etc.*) against some cellular targets (*e.g*., mitochondria, RER) and/or lysosomal damage/dysfunction. This finally results in increased ROS levels, apoptosis, DNA and membrane damage.

Notably, the cellular toxicity will also depend on the physico-chemical characteristics of the NPs (such as the size, nature and chemical stability of the core and ligands, surface coating, *etc.*), as their uptake mechanism and subsequent corrosion behavior will be a strong function of these characteristics. Also, such variables, together with protein corona effects, will affect NP uptake and their subsequent sub-cellular localization^18,27^ so that NPs may encounter different microenvironments and different pHs, which, in turn, will modulate the rate of NPs degradation (and thus their toxicity). In some cases, for instance, less acidic pHs in the endo/lysosomal pathway may slow down NP degradation, mitigating the toxic effects. Such a mechanism partially accounts for the dependence of cytotoxicity on the experimental conditions (*e.g.*, culture medium, which affects protein corona formation) as well as on the specific cell line.

A result of our study is that, in principle, it can be envisaged that the slower the lysosomal degradation of the NPs, the lower their cellular toxicity. As a consequence, we decided to apply our model to identify, by design, biocompatible nanoparticles. We selected, as a first example, our Fe_3_O_4_ NPs. By passivating the surface of the thin silica shell surrounding the Fe_3_O_4_ NPs (by functionalization with 3-(trihydroxysilyl)-1-propanesulfonic acid), we were able to reduce the ion leakage from the NPs in acidic conditions (Fig. S27A†), while maintaining the original characteristics of the NPs (same size, same surface charge (–28 ± 5 mV), same cellular internalization). Interestingly, such a procedure results in a remarkable reduction of cellular toxicity (Fig. S27B†) as compared to the non-passivated NPs. Hence, the very same NP was found to be largely less toxic because its suitable surface engineering strongly reduces its intracellular release of toxic ions. Furthermore, we tested QDs made with a different metallic core (*i.e.*, indium instead of cadmium), finding a similar particle degradation in acidic, lysosomal-like conditions, but no detectable impact on cellular viability or membrane integrity (Fig. S28†), as indium ions are significantly better tolerated by cells than cadmium. Moreover, as additional examples, we investigated particles that do not corrode or degrade into biocompatible components. As an example for the former, we chose diamond nanoparticles (NDs) and for the latter we chose silica particles (SiO_2_ NPs). We used 5–10 nm nanodiamonds and found no measurable toxicity even using a significantly higher dose range (see Fig. S29†), in agreement with the recent literature.^50,51^ We then moved to amorphous SiO_2_ NPs that, though internalized in cells *via* active mechanisms, were demonstrated to be non-toxic to several cell lines^52^ and *in vivo*.^53^ Consistently, we found that SiO_2_ NPs are quite resistant to acidic conditions, in which they release a low amount of silicic acid (see also Fig. S30†).^53^ This species is naturally found in numerous tissues and is known to be a non-toxic compound;^53–55^ moreover, silicic acid administered to humans is efficiently excreted from the body through the urine.^53^ As a final example, we tested ceria NPs, finding no appreciable cell toxicity in the tested concentration range (Fig. S31†). Indeed, ceria NPs have been proposed as potential candidates to fight chronic inflammation and oxidative stress, thanks to their significant antioxidant properties. It seems that nanoceria can mimic the behavior of two key antioxidant enzymes (superoxide dismutase and catalase), likely eliminating intracellular ROS *via* a self-regenerating mechanism.^56^ Representative confocal microscopy images of lysosomal co-localization for some of the tested NPs are reported in Fig. S23–S26.† Overall, the LETH toxicity model opens up several routes for the development of suitable surface ligands or coatings that can prevent/reduce NP toxicity.

All the experiments performed in this work and the relative findings have been summarized in Table S37 in the ESI.†

In conclusion, we have shown that, for a wide range of NPs (metallic, oxide, and semiconductor), the primary intracellular toxicity mechanism is common and can be explained in terms of the LETH model. Since the main cause of toxicity induced by metal containing NPs is ascribed to their corrosion in the acidic lysosomal environment, it is possible to envisage several strategies to engineer safer NPs, while maintaining their characteristic and powerful functionalities. Among such promising concepts are: (i) design of specific NP coatings to allow NPs to skip endocytotic processes; (ii) design of specific ligands or bioconjugation procedures that allow for a rapid lysosomal escape;^57,58^ (iii) development of robust and stable surface coatings that are resistant to the acidic pH of lysosomes and prevent metal ion leakage. Notably, the first two strategies are particularly attractive for nanomedicine/drug delivery applications as they include the additional possibility of a direct release of the NP cargo into the cell cytosol. On the other side, the third approach provides a general route, of potential industrial interest, towards the realization of biocompatible metal containing NPs. Moreover, although the LETH toxicity model described in this work cannot account for all the toxicity issues (*e.g.*, in the case of different uptake routes of NPs such as inhalation, in the case of NP-induced inflammation,^7^ or in the case of environmentally dispersed NPs), it may constitute a first step toward a more detailed knowledge and categorization of the toxicity mechanisms of several classes of engineered NPs. This would also pave the way towards the development of safer nanoparticles by design and LETH-based strategies to eliminate the toxicity of metal containing NPs.
