# Supplementary material for: A general mechanism for intracellular toxicity of metal-containing nanoparticles
Source: Nanoscale. 2014 May 20;6(12):7052–61. doi: 10.1039/c4nr01234h (PMC4120234; doi:10.1039/c4nr01234h)
Supplement: Supplementary file 1 [file NR-006-C4NR01234H-s001.pdf]

Electronic Supplementary Material (ESI) for Nanoscale.
This journal is © The Royal Society of Chemistry 2014

Supplementary Information

A General Mechanism for Intracellular Toxicity of Metal-Containing Nanoparticles

Stefania Sabella[1], Randy Carney[2], Virgilio Brunetti[1], Maria Ada Malvindi[1], Noura Al-Juffali[2,3], Giuseppe Vecchio[1], Sam M. Janes[3], Osman M. Bakr[4], Roberto Cingolani[5], Francesco Stellacci[2]*, and Pier Paolo Pompa[1]*

[1] Istituto Italiano di Tecnologia, Center for Bio-Molecular Nanotechnologies@UniLe, Via Barsanti – 73010 Arnesano (Lecce), Italy

[2] Institute of Materials, École Polytechnique Fédérale de Lausanne (EPFL), CH-1015 Lausanne, Switzerland

[3] Centre For Respiratory Research, Rayne Institute, University College London, 5 University Street, London WC1E 6JJ, UK

[4] Division of Physical Sciences and Engineering, Solar and Photovoltaics Engineering Center, King Abdullah University of Science and Technology (KAUST), Thuwal 23955- 6900, Saudi Arabia

[5] Istituto Italiano di Tecnologia, Central Research Laboratories, Via Morego, 30 – 16136 Genova, Italy

*To whom correspondence should be addressed. E-mails: pierpaolo.pompa@iit.it; francesco.stellacci@epfl.ch

This file includes:
Figs S1 to S36
Tables S32, S33, S37
Materials and Methods
References

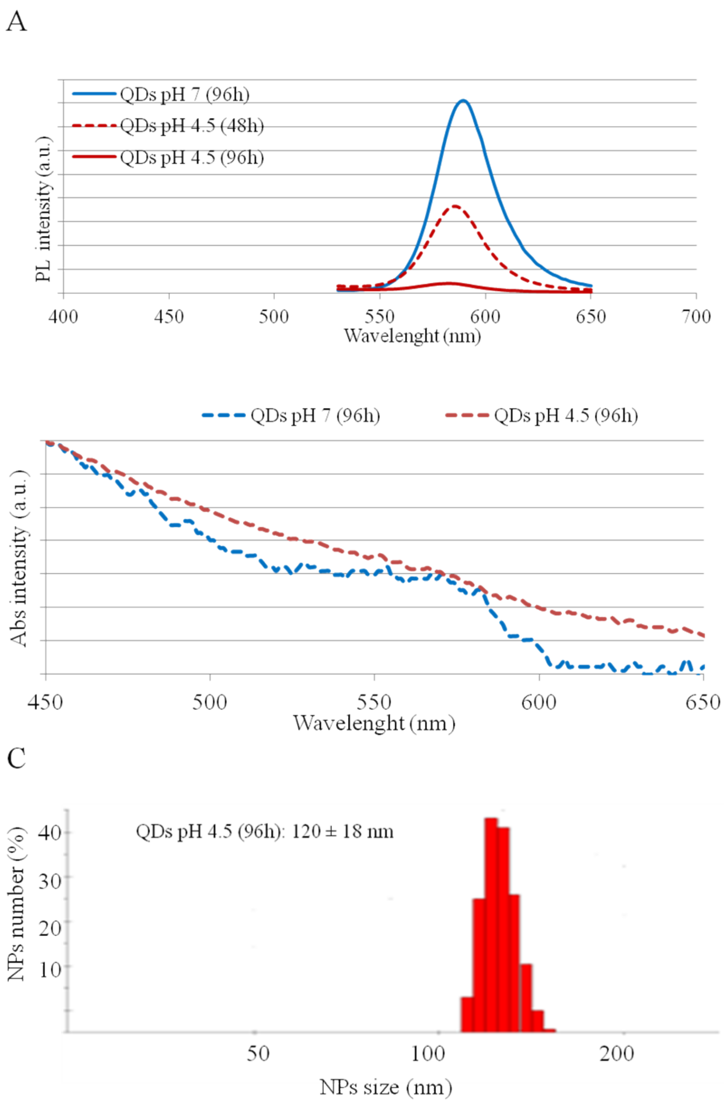

Fig. S1. Behavior of CdSe/ZnS QDs in neutral (pH 7, 37 °C) and acidic conditions (pH 4.5, 37 °C) over time. (A) Photoluminescence spectra (the reference PL spectrum at time 0 completely overlaps with the blue curve and is not shown for clarity); (B) Absorption spectra; (C) DLS measurement of QDs after 96 h incubation in acidic conditions.

As shown, CdSe/ZnS QDs, when incubated in acidic conditions, gradually but significantly lose their fluorescence property, due to particle core degradation, unlike QDs in neutral environment that, after 96 h, completely maintain their original photo-physical properties (in terms of both PL spectrum and emission intensity). Such behavior is also confirmed by absorption measurements, revealing that incubation in the acidic environment results in the loss of the QDs excitonic peak. Furthermore, the QDs in the acidic conditions degrade and became significantly less stable in solution, showing significant precipitation and presence of aggregates (panel C).

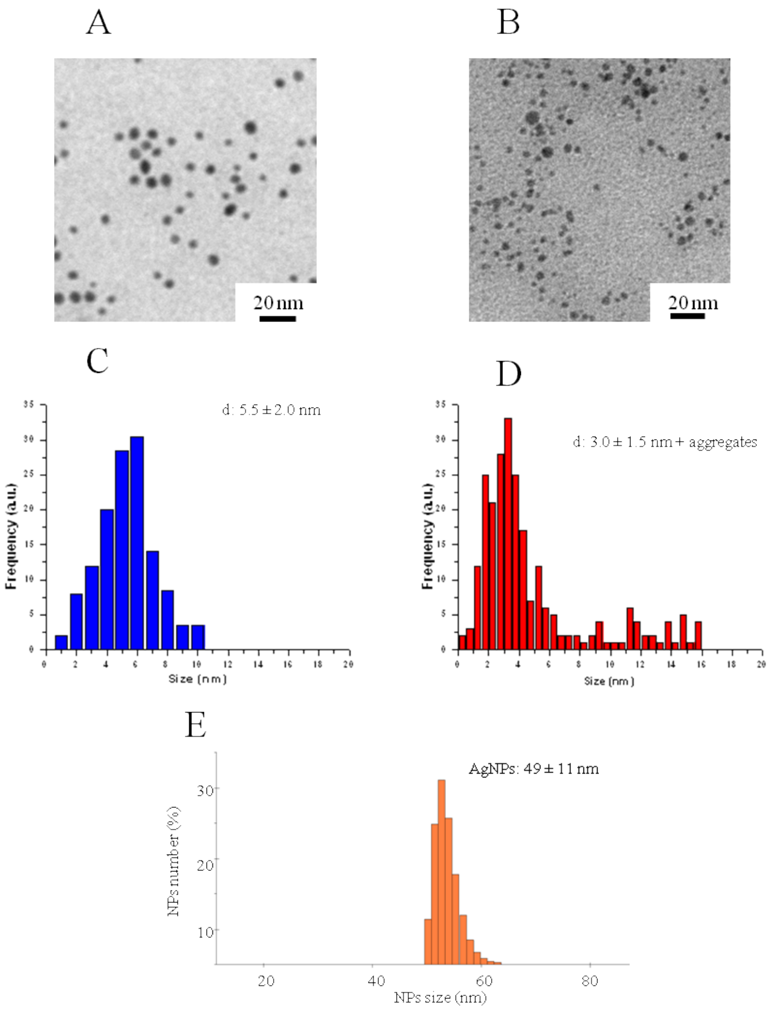

Fig. S2. Behavior of AgNPs in neutral (pH 7, 37 °C) and acidic conditions (pH 4.5, 37 °C) over time. (A, B) Representative TEM images of AgNPs after 96 h incubation in (A) neutral conditions and (B) in acidic environment. (C, D) TEM size distributions of AgNPs in the conditions of A and B, respectively. (E) DLS measurement of AgNPs after 96 h incubation in acidic conditions.

It is evident that the lysosomal-like environment strongly promotes NP corrosion and ion release, unlike neutral (cytoplasmic-like) conditions. In particular, TEM imaging clearly reveals that incubation in acidic conditions results in strong NP degradation, with the formation of smaller nanoparticles (see panels C and D). The presence of some aggregates is also visible. AgNP degradation in acidic conditions is revealed also by DLS analyses, which show significant NP aggregation in solution.

It should be clarified that the findings of the ion release experiments are that NP degradation and ion release are much faster in acidic conditions than in neutral conditions, namely the acidic (lysosomal-like) conditions strongly promote NP dissolution. This means that it may be possible to detect ion release also in neutral conditions (for instance, by increasing the NP concentration or the detection sensitivity) but, in any case, the acidic environment will promote significantly higher release. For example, in the case of AgNPs, there is indeed some ion release also in neutral conditions (this is the basis of their antibacterial behavior), but in acidic conditions such process is significantly higher[1].

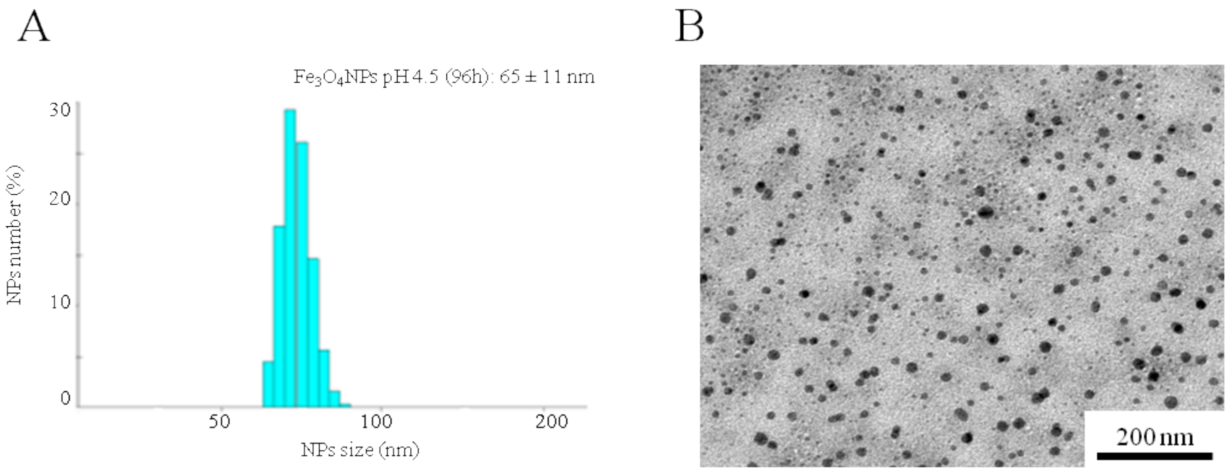

Fig. S3. Degradation of Fe_3O_4 NPs in acidic conditions (pH 7, 37 °C). (A) DLS measurement after 96 h incubation at pH 4.5; (B) TEM image of NPs after 20 days incubation in acidic conditions. When incubated in acid environment, Fe_3O_4 NPs undergo significant degradation, releasing iron ions and losing their magnetic properties (see also Fig. 1). Moreover, they show a strong decrease of their stability in solution, presenting large NPs precipitates, detectable by naked eye, and extensive aggregation (panel A). The acidic corrosion of these NPs can be clearly appreciated by prolonging the incubation time in acidic environment up to 20 days. In these conditions, in fact, TEM analyses reveals large NP dissolution (panel B), with the massive presence of small NP fragments.

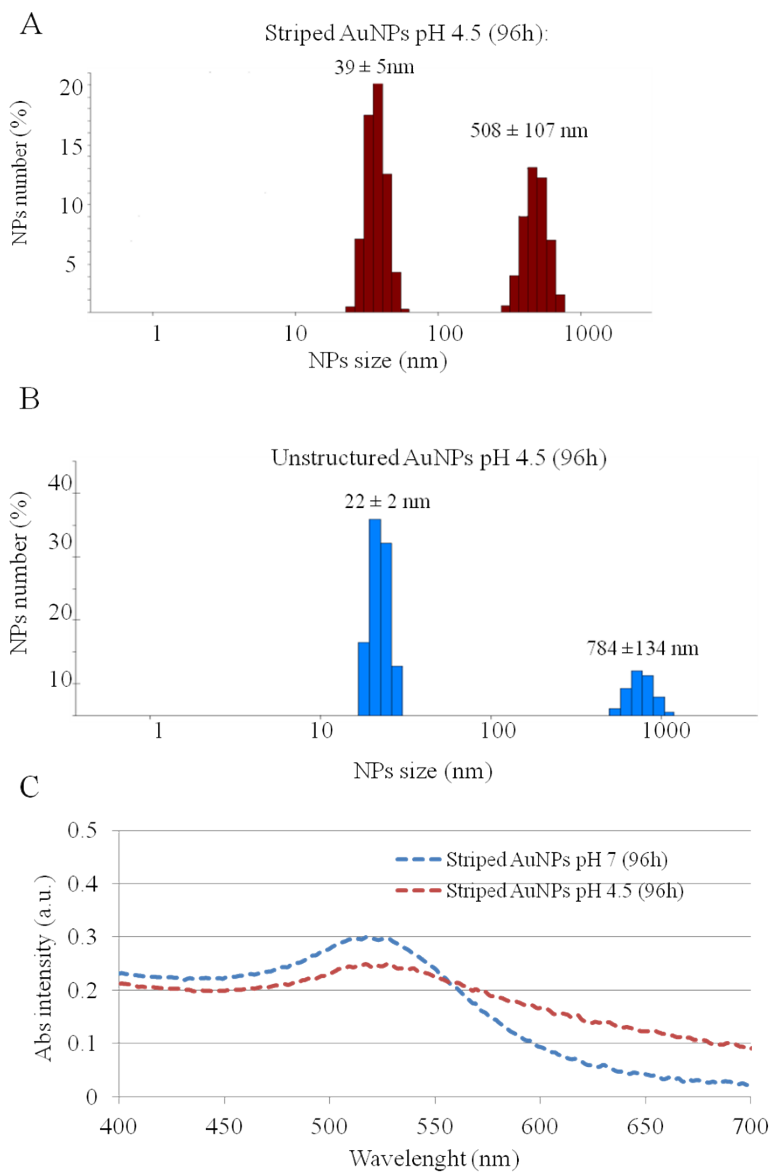

Fig. S4. Behavior of AuNPs upon incubation in neutral (pH 7, 37 °C) and acidic conditions (pH 4.5, 37 °C). (A, B) DLS measurements of striped and unstructured AuNPs after 96 h incubation in acidic conditions. (C) Absorption spectra of striped AuNPs after 96 h incubation in neutral and acidic conditions.

In the case of gold, the NP corrosion and ion release is lower (see also Fig. 1), so it can be appreciated only as a significant loss of their stability in solution with consequent NP aggregation. Such phenomenon is evident for both striped and unstructured AuNPs in both DLS and absorption experiments.

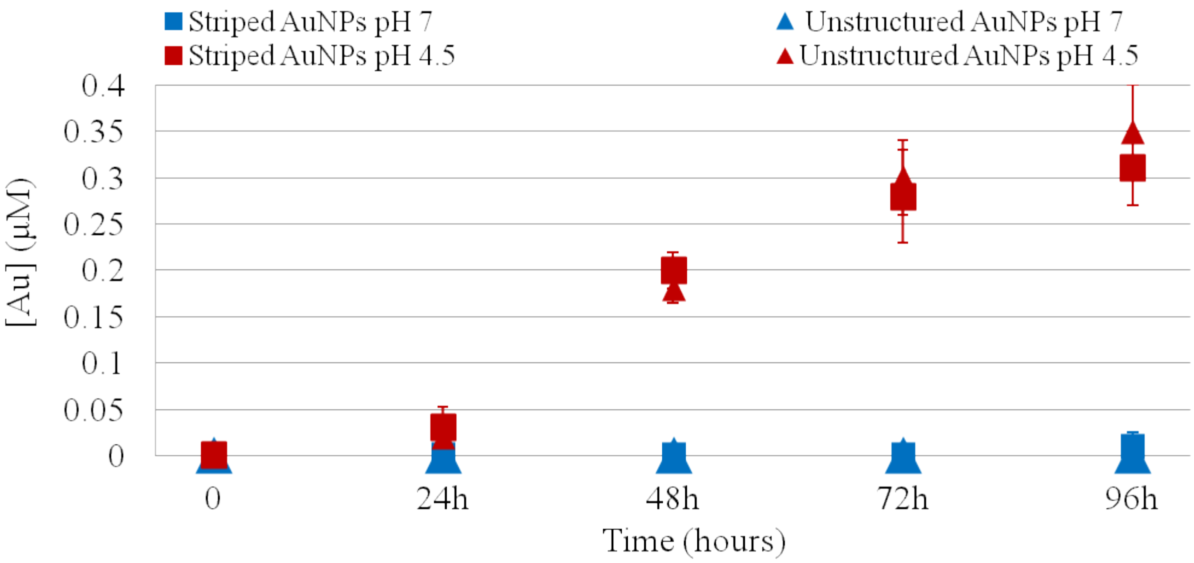

Fig. S5. Time-dependent gold ion release from striped and unstructured AuNPs (50 nM), probed by ICP-AES, at 37 °C, at neutral (pH 7.0, blue symbols) or acidic (pH 4.5, red symbols) conditions. Data represent the average from 3 independent measurements (6 replicates for each experiment) and the error bars indicate the standard deviation. The two AuNPs release comparable amounts of gold ions in acidic conditions, while at neutral pH the gold ion release was not detectable.

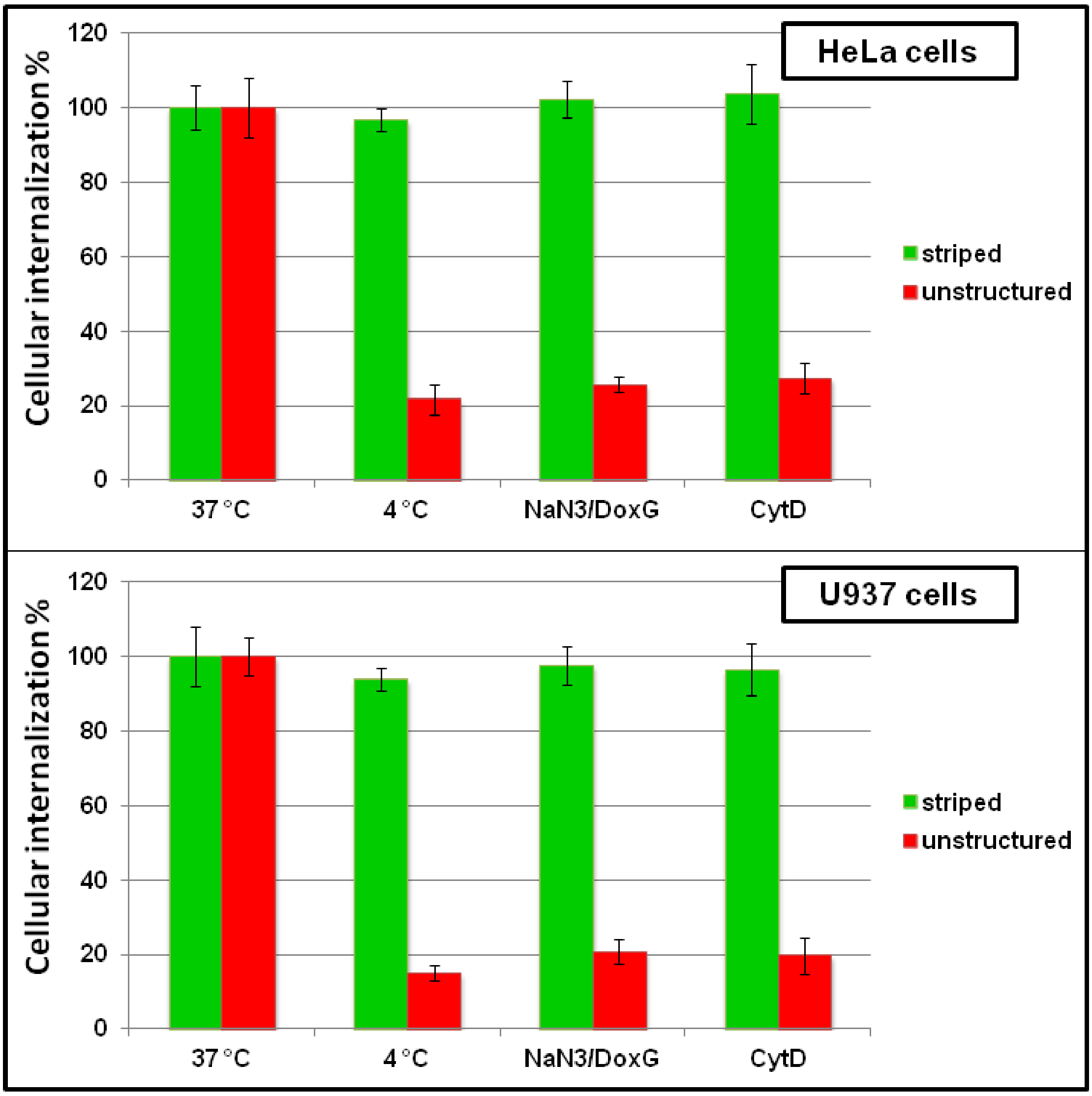

Fig. S6. ICP-AES analyses of the amount of AuNPs internalized by U937 and HeLa cells (normalized to the control at 37 °C) in the presence of specific inhibitors of endocytic processes, namely 4 °C, sodium azide/2-deoxyglucose (NaN3/DoxG), Cytochalasin D (CytD), confirming that the striped AuNPs are able to penetrate the cells regardless of the activation/inhibition of the active energy-dependent internalization processes, unlike unstructured AuNPs.

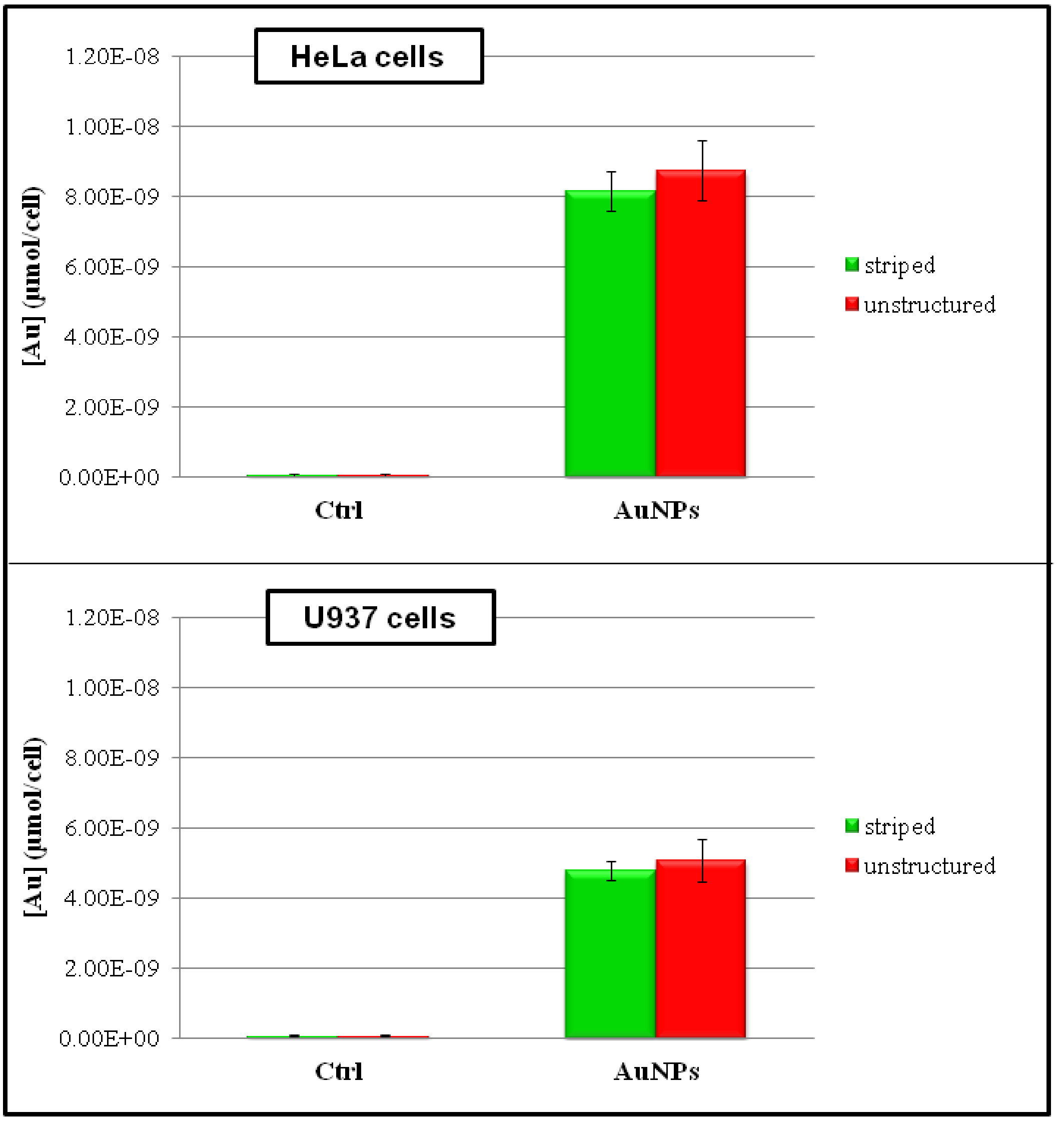

Fig. S7. Cellular internalization of striped and unstructured AuNPs probed by ICP-AES. HeLa and U937 were treated with striped and unstructured AuNPs (20 nM) for 48 hours. After incubation, cells were washed and analyzed as reported in Methods. Results indicate that the two AuNPs are taken up in a similar manner by both cell lines.

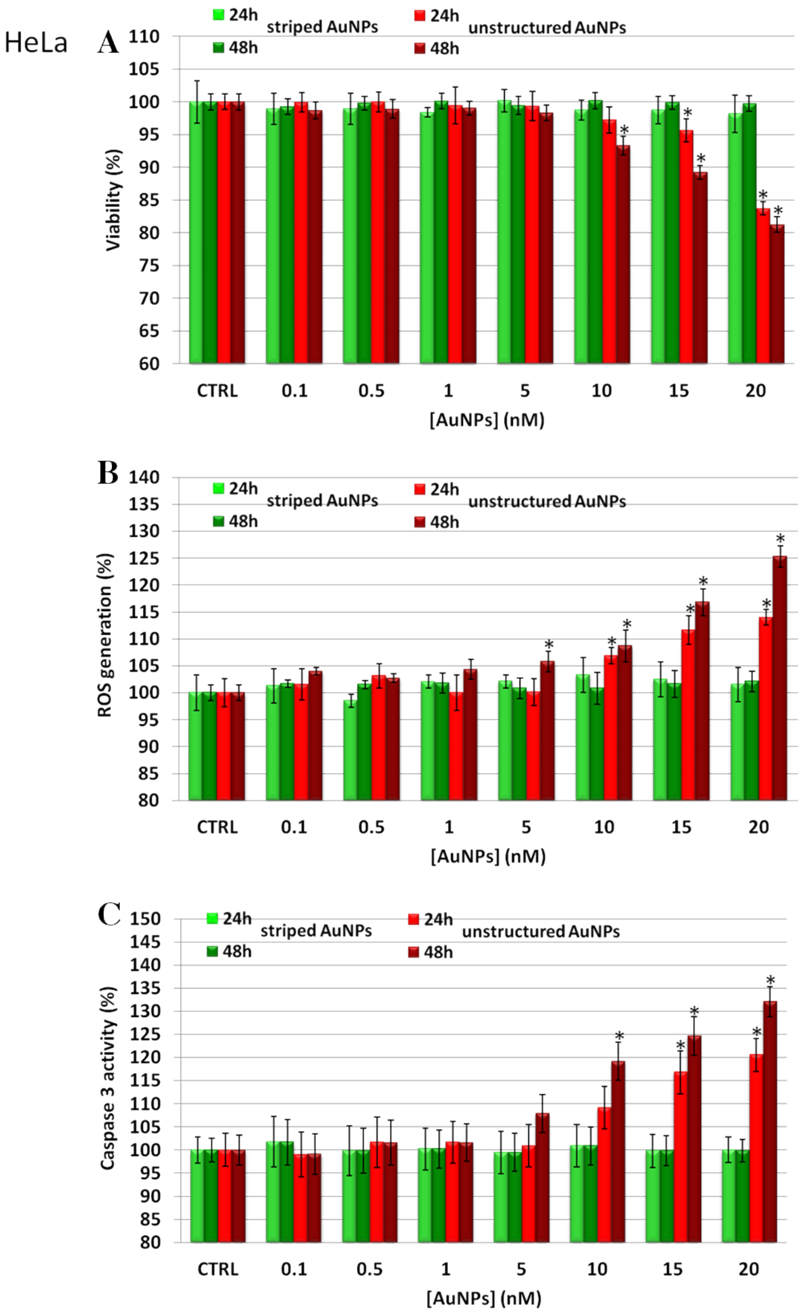

Fig. S8. Toxicity assessment of striped and unstructured AuNPs in human cervix carcinoma, epithelial cells (HeLa). (A) WST-8 proliferation assay upon treatment with increasing amount of AuNPs. CTRL represents the negative control; values are means+s.d. Positive controls (not shown) were treated with 0.01% of TritonX100, displaying a viability decrease (ca. 85-95%) with respect to the untreated cells. (B) ROS quantification, via DCFH-DA assay, after cellular treatment with AuNPs; values are means+s.d. Positive controls (not shown) were treated with a free radical generator (100 µM H_2O_2), exhibiting a ROS increase of ca. 200-220% with respect to the untreated control cells. (C) Evaluation of Caspase 3 activity. Values are means+s.d. Results were analyzed by Two-way ANOVA and values compared to the control by Bonferroni post-hoc test. Differences between treated samples and controls (n=8) were considered statistically significant for *P<0.05, non significant for P>0.05.

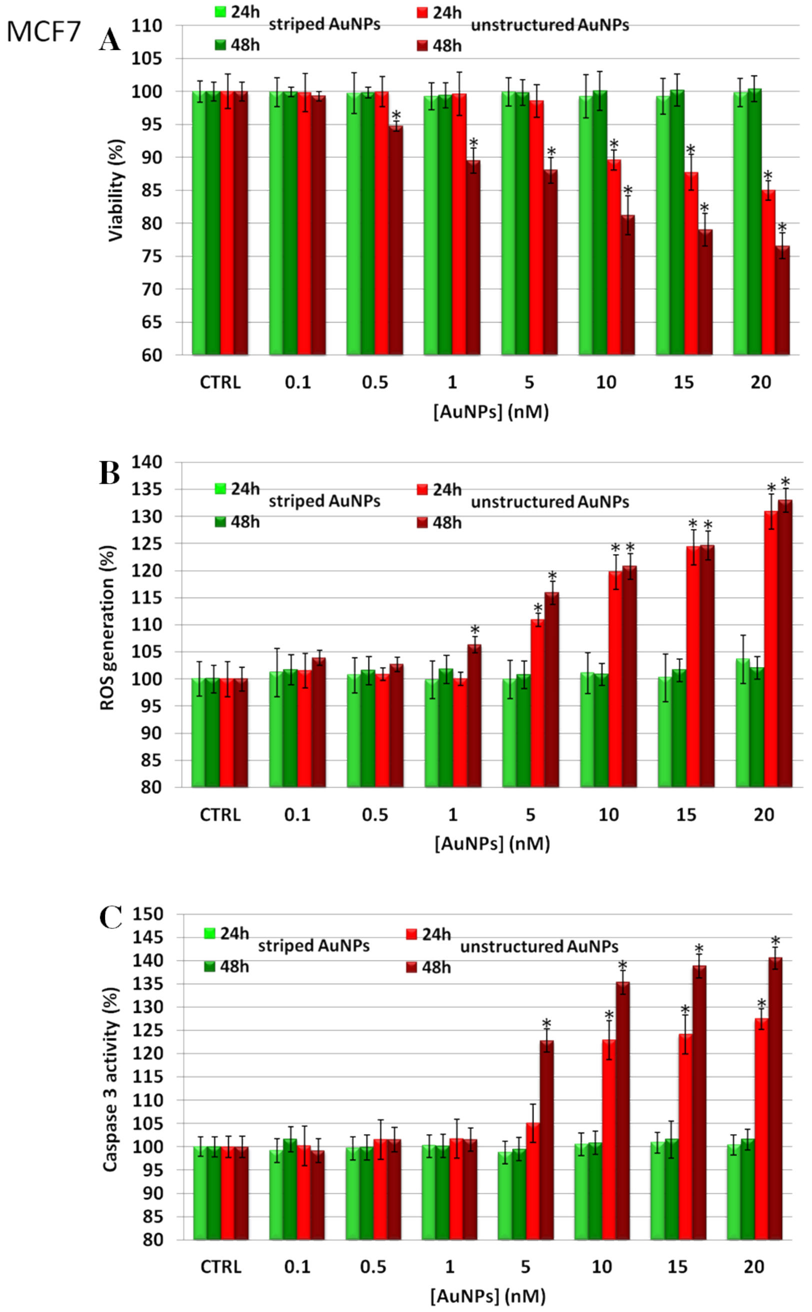

Fig. S9. Toxicity assessment of striped and unstructured AuNPs in human breast adenocarcinoma epithelial cells (MCF7). (A) WST-8 proliferation assay upon treatment with increasing amount of AuNPs. CTRL represents the negative control; values are means+s.d. Positive controls (not shown) were treated with 0.01% of TritonX100, displaying a viability decrease (ca. 80-90%) with respect to the untreated cells. (B) ROS quantification, via DCFH-DA assay, after cellular treatment with AuNPs; values are means+s.d. Positive controls (not shown) were treated with a free radical generator (100 µM H_2O_2), exhibiting a ROS increase of ca. 180-190% with respect to the untreated control cells. (C) Evaluation of Caspase 3 activity. Values are means+s.d. Results were analyzed by Two-way ANOVA and values compared to the control by Bonferroni post-hoc test. Differences between treated samples and controls (n=8) were considered statistically significant for *P<0.05, non significant for P>0.05.

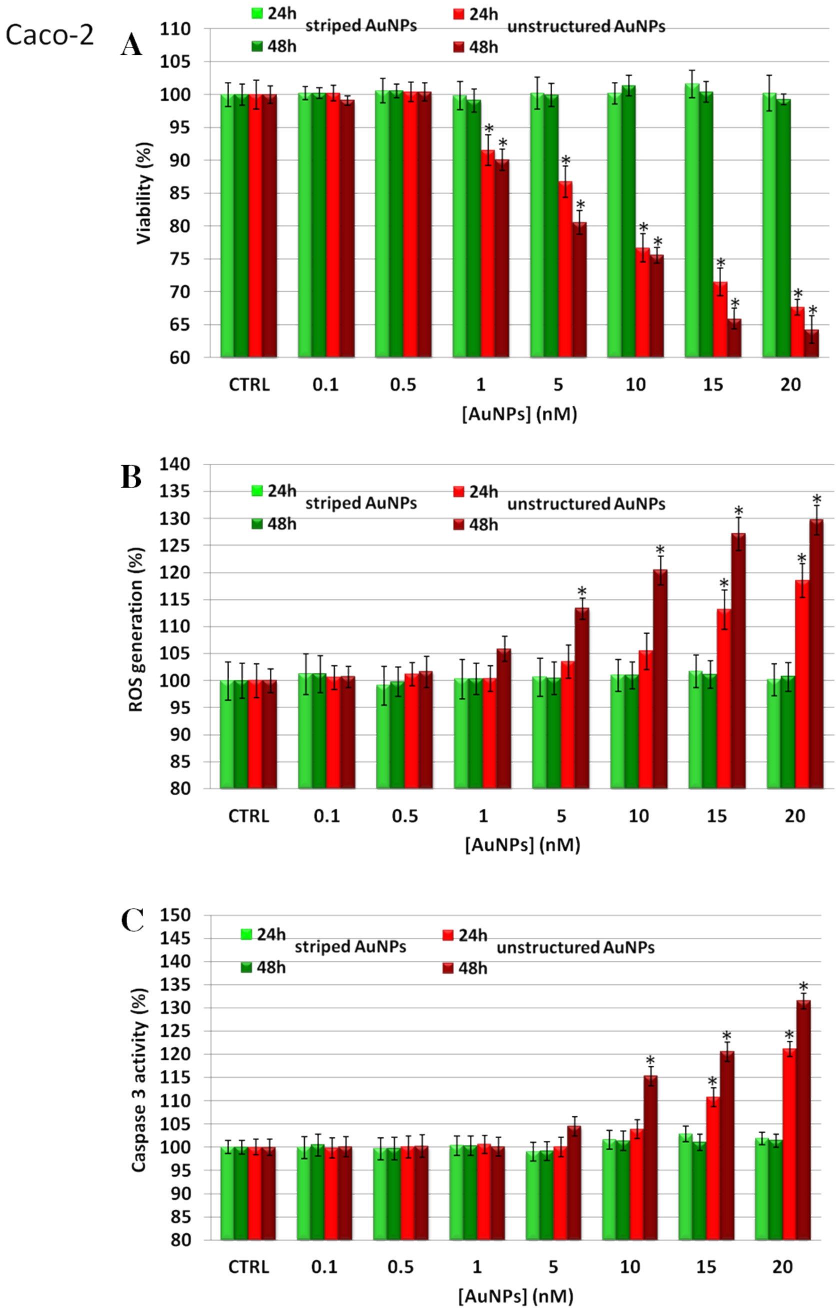

Fig. S10. Toxicity assessment of striped and unstructured AuNPs in human colon adenocarcinoma epithelial cells (Caco-2). (A) WST-8 proliferation assay upon treatment with increasing amount of AuNPs. CTRL represents the negative control; values are means+s.d. Positive controls (not shown) were treated with 0.01% of TritonX100, displaying a viability decrease (ca. 80-90%) with respect to the untreated cells. (B) ROS quantification, via DCFH-DA assay, after cellular treatment with AuNPs; values are means+s.d. Positive controls (not shown) were treated with a free radical generator (100 μM H_2O_2), exhibiting a ROS increase of ca. 180-190% with respect to the untreated control cells. (C) Evaluation of Caspase 3 activity. Values are means+s.d. Results were analyzed by Two-way ANOVA and values compared to the control by Bonferroni post-hoc test. Differences between treated samples and controls (n=8) were considered statistically significant for *P<0.05, non significant for P>0.05.

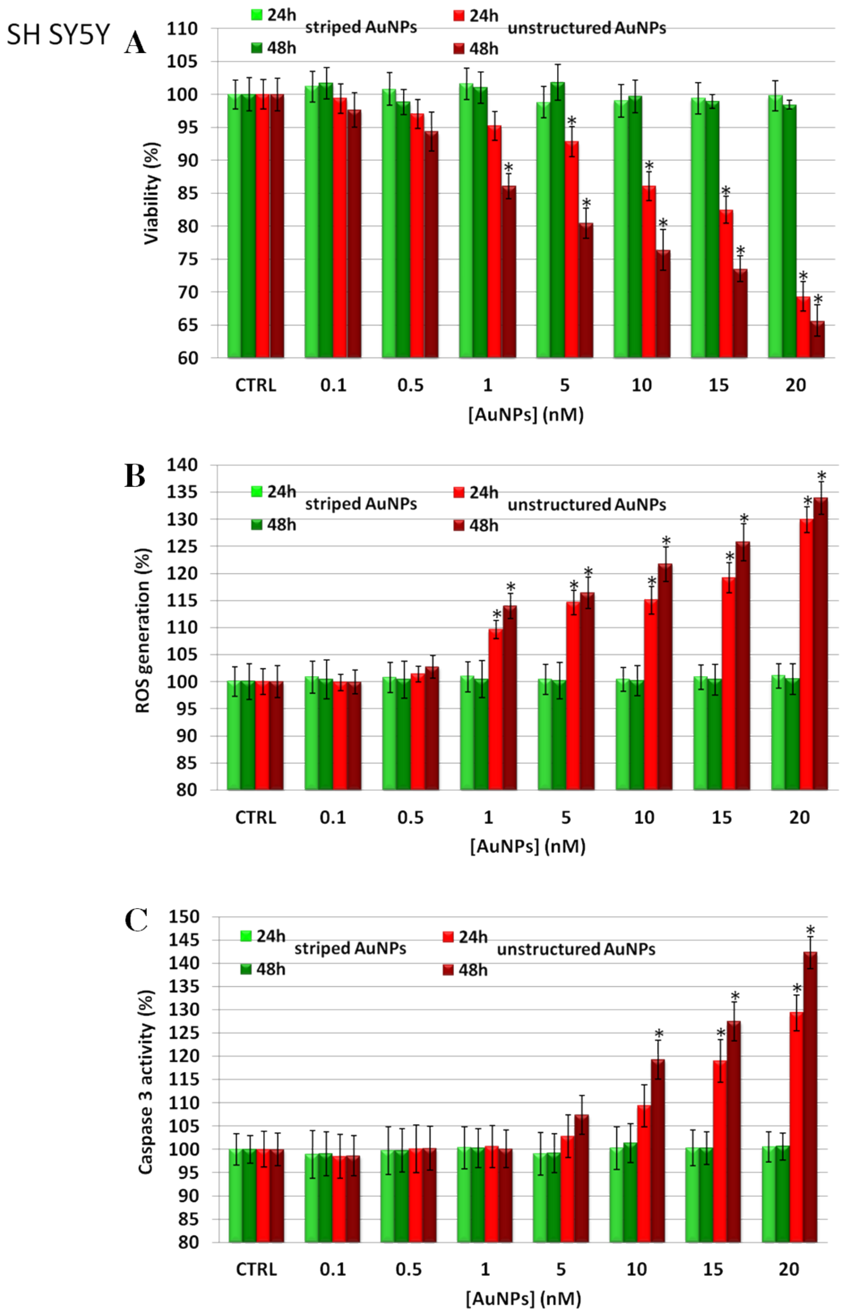

Fig. S11. Toxicity assessment of striped and unstructured AuNPs in human neuroblastoma cells (SH SY5Y). (A) WST-8 proliferation assay upon treatment with increasing amount of AuNPs. CTRL represents the negative control; values are means+s.d. Positive controls (not shown) were treated with 0.01% of TritonX100, displaying a viability decrease (ca. 90-95%) with respect to the untreated cells. (B) ROS quantification, via DCFH-DA assay, after cellular treatment with AuNPs; values are means+s.d. Positive controls (not shown) were treated with a free radical generator (100 μM H_2O_2), exhibiting a ROS increase of ca. 200-210% with respect to the untreated control cells. (C) Evaluation of Caspase 3 activity. Values are means+s.d. Results were analyzed by Two-way ANOVA and values compared to the control by Bonferroni post-hoc test. Differences between treated samples and controls (n=8) were considered statistically significant for *P<0.05, non significant for P>0.05.

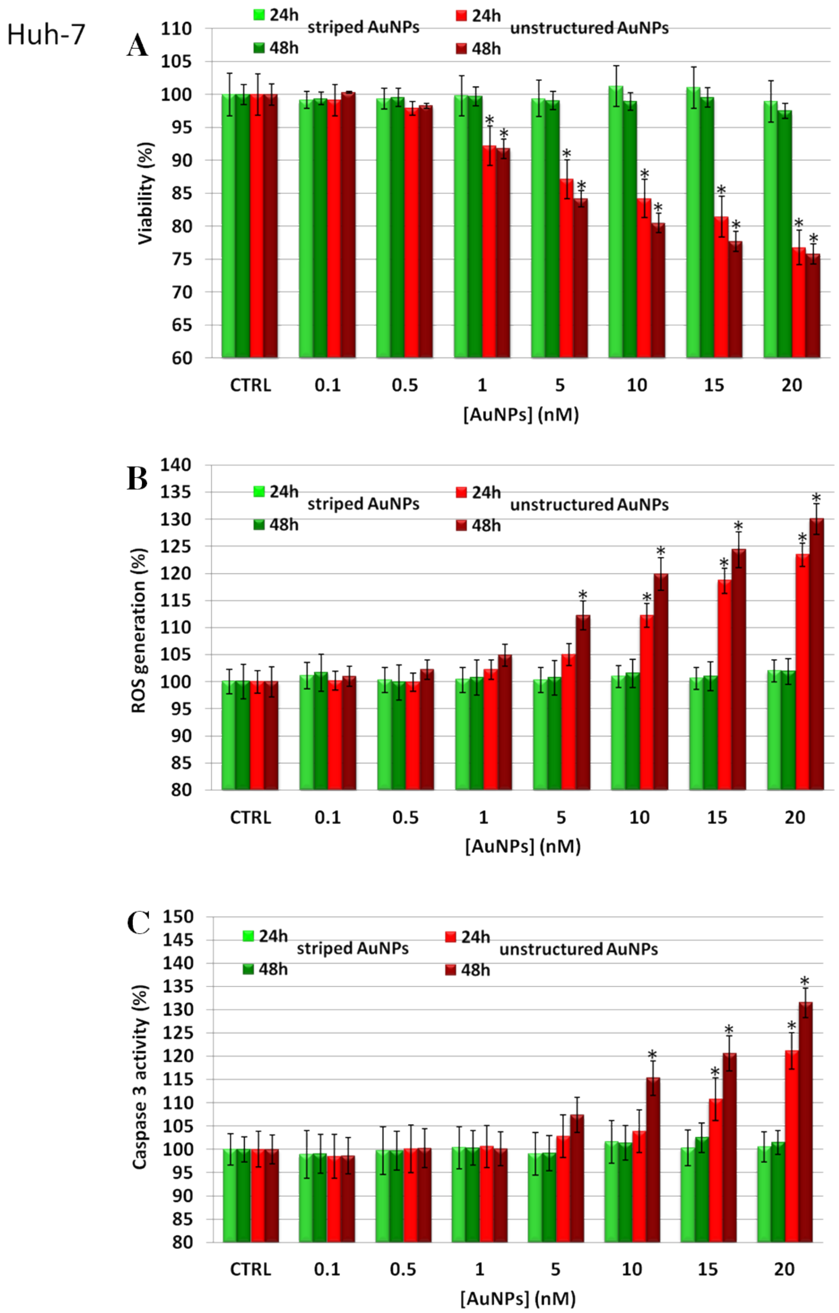

Fig. S12. Toxicity assessment of striped and unstructured AuNPs in human hepatoma cells (Huh-7). (A) WST-8 proliferation assay upon treatment with increasing amount of AuNPs. CTRL represents the negative control; values are means+s.d. Positive controls (not shown) were treated with 0.01% of TritonX100, displaying a viability decrease (ca. 85-90%) with respect to the untreated cells. (B) ROS quantification, via DCFH-DA assay, after cellular treatment with AuNPs; values are means+s.d. Positive controls (not shown) were treated with a free radical generator (100 µM H_2O_2), exhibiting a ROS increase of ca. 190-200% with respect to the untreated control cells. (C) Evaluation of Caspase 3 activity. Values are means+s.d. Results were analyzed by Two-way ANOVA and values compared to the control by Bonferroni post-hoc test. Differences between treated samples and controls (n=8) were considered statistically significant for *P<0.05, non significant for P>0.05.

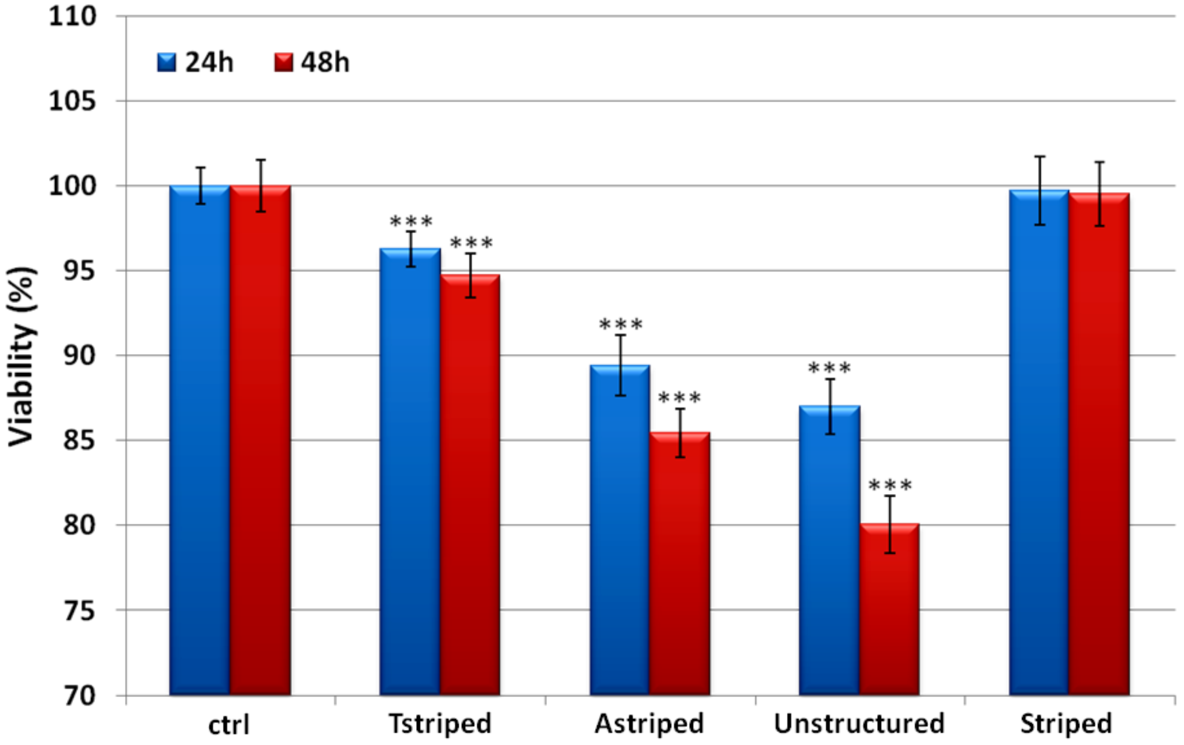

Fig. S13. Viability assay in U937 cells of unstructured and striped AuNPs, compared to transferrin- or apolipoprotein-conjugated striped NPs (indicated as Tstriped and Astriped, respectively). AuNPs concentration was 65 nM. Ctrl represents the negative control; values are means+s.d.. Results were analyzed by Two-way ANOVA and values compared to the control by Bonferroni post-hoc test. Differences between treated samples and controls (n=8) were considered statistically significant for ***P<0.001.

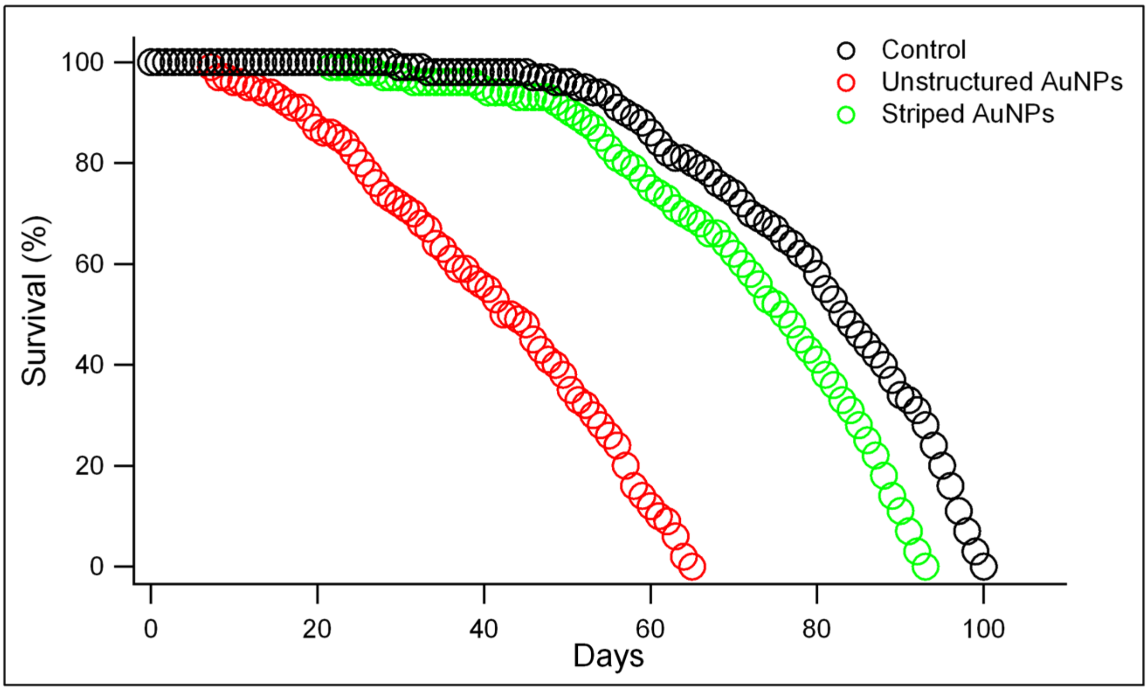

Fig. S14. Lifespan curves of *Drosophila* flies nurtured with unstructured and striped AuNPs treated food (dose: 0.36 µg/g per day) compared to the population bred with normal food (control). Experimental points represent the average from 5 independent experiments and the error bars (reported as diameter of points) indicate the standard deviation. The lifespan curves were validated by the non-parametric log-rank (Mantel-Cox) test followed by Gehan-Breslow-Wilcoxom post-test (CTRL *vs* unstructured AuNPs: $p<0.0001$; CTRL vs striped AuNPs: not significant (ns)).

A significant lifespan reduction was induced by the treatment with the unstructured particles (τ_{50} was ca. 47% lower than the control), as opposed to striped particles, despite a similar bioaccumulation of the two AuNPs in the organisms (ca. 0.25 pg of gold/organism were detected by ICP-AES after 30 days of treatment with both AuNPs).

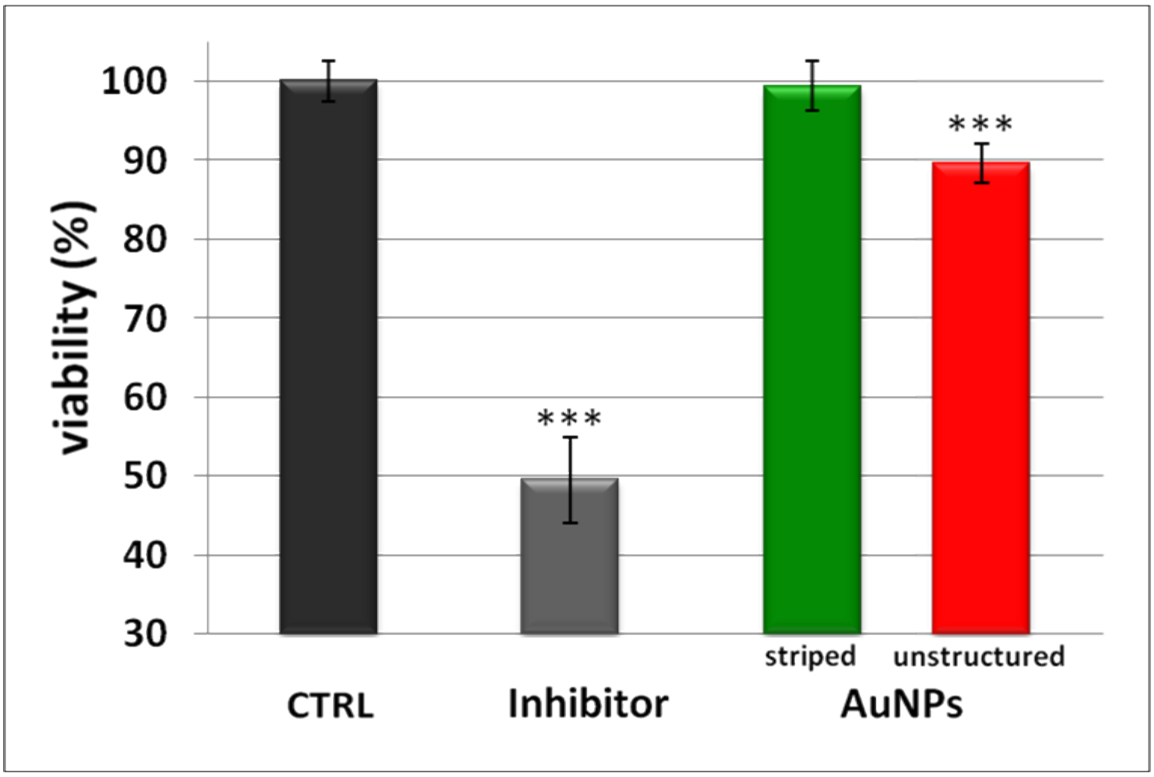

Fig. S15. Viability test of HeLa cells by WST-8 assay upon treatment with striped and unstructured AuNPs at the same experimental conditions employed for the TrxR inhibition assay showed in Fig. 4. HeLa cells were treated with 15 nM of striped and unstructured AuNPs and 1 µM of inhibitor (Auranofin) for 48 h. Ctrl represents the negative control; values are means+s.d. and differences between treated samples and controls (n=8) were considered statistically significant for ***P<0.001.

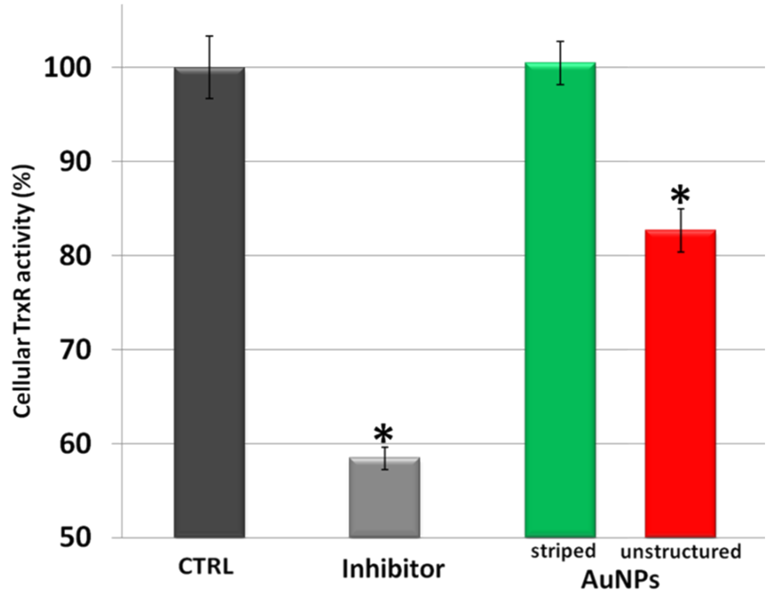

Fig. S16. Cellular TrxR activity in U937 cells after 48 h treatment with striped and unstructured AuNPs (15 nM) and Auranofin (1 μM) (see Methods for experimental details). Results are mean ± SD and differences between treated samples and controls (n=8) were considered statistically significant for *P<0.05, non significant for P>0.05.

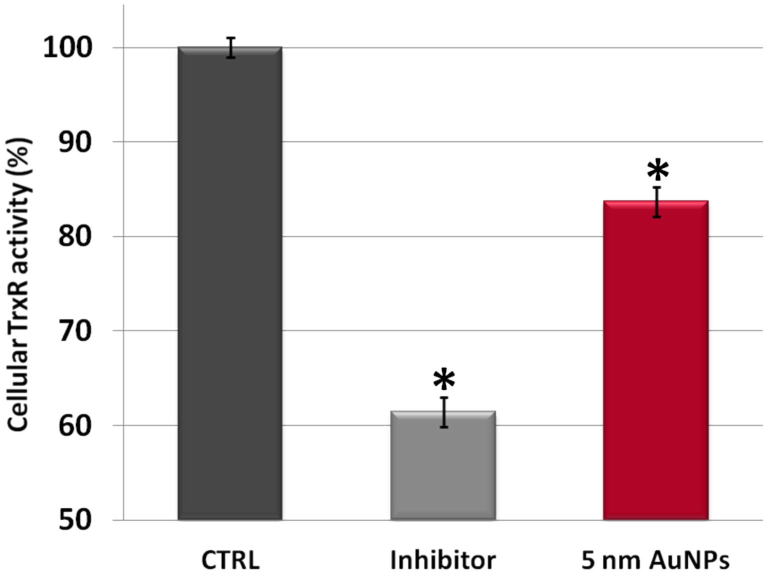

Fig. S17. Cellular TrxR activity in HeLa cells after 48 h treatment with 5 nm citrate capped AuNPs (15 nM) and Auranofin (1 μM) (see Methods for experimental details). Results are mean ± SD and differences between treated samples and controls (n=8) were considered statistically significant for *P<0.05, non significant for P>0.05.

Mammalian thioredoxin reductase (TrxR): interactions with gold based compounds

The pharmaceutical chemistry of gold ions and of their relative complexes used as therapeutic agents has been extensively studied due to their extensive pharmacological use as anti-rheumatic drugs[2]. Moreover, many gold-based drugs (including solganol, myocrisin, auranofin, etc.) have recently gained great attention as very potent and specific blockers of the enzyme thioredoxin reductase (TrxR)[3]. Interestingly, due to the principal function of TrxRs family to reduce oxidized thioredoxin (Trx)[4,5], the blocking of this enzyme impacts the regulation of many cell functions under Trxs control, such as regulation of the redox signaling pathways, antioxidant activity (by removing the hydrogen peroxides through peroxiredoxin), control of transcription factors binding, and inhibition of apoptosis[6]. In particular, when mitochondrial TrxR is damaged, the thiol redox balance is altered, thus triggering the apoptosis *via* swelling or improved permeabilization of the mitochondrial outer membrane[7,8]. Such event is the “point of no return”, since it primes the cascade release of many pro-apoptotic signaling molecules, including cytochrome *c* and caspases[9,10]. Moreover, the TrxR inactivation does not lead to the normal recycling activity of ROS (e.g., the hydrogen peroxide mainly formed by the mitochondrial respiratory chain[6] thus contributing to an overall alteration of the ROS basal levels in the cells[11].

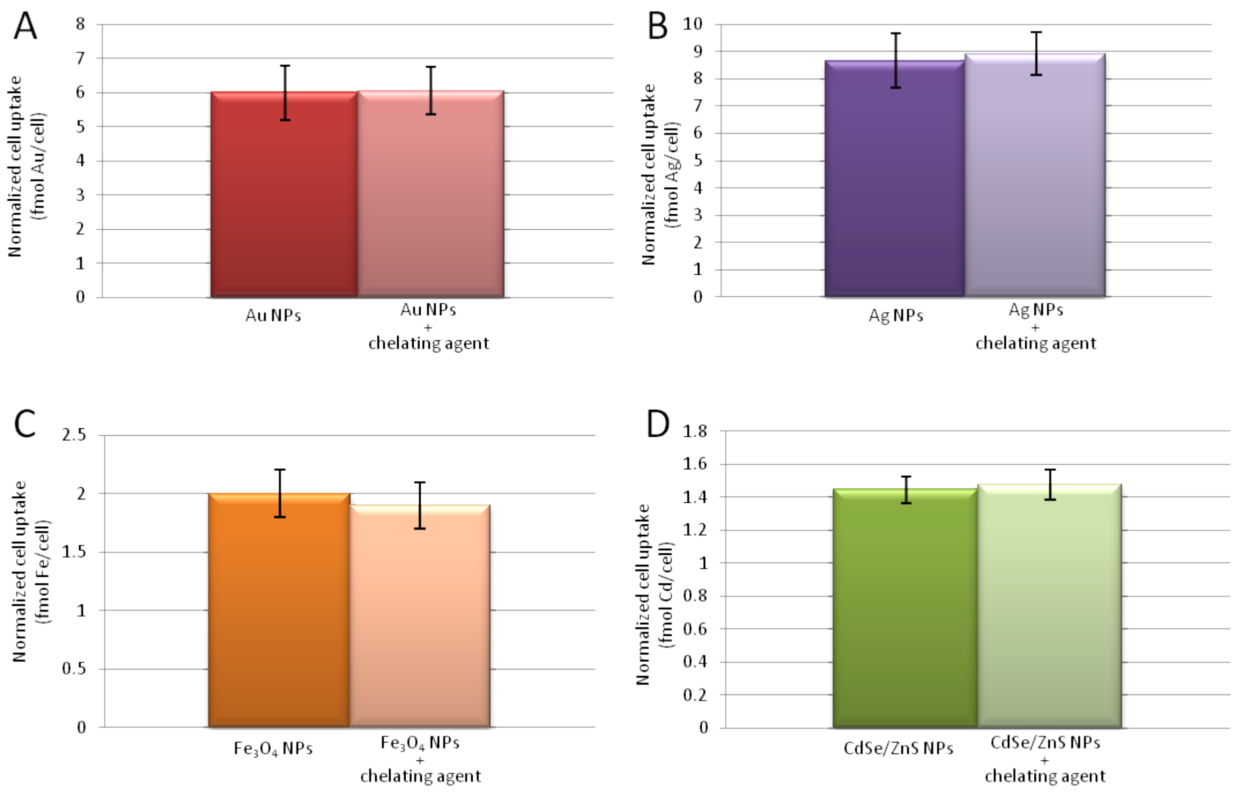

Fig. S18. Cellular uptake of different NPs in presence or absence of chelating agents. (A) unstructured AuNPs (20 nM); (B) AgNPs (2 nM); (C) Fe_3O_4 NPs (2.5 nM); CdSe/ZnS QDs (5 nM). HeLa cells were pretreated for 30 min with/without chelating agents (A,B,D: BAL; C: dfx) and then exposed to the NPs for 24 h. It is clear that the cellular uptake of the different NPs is not influenced by the chelating agents. Values are means+s.d (n=8). The negative controls (not shown), representing cells without NPs treatment, all exhibited non-detectable metal amounts.

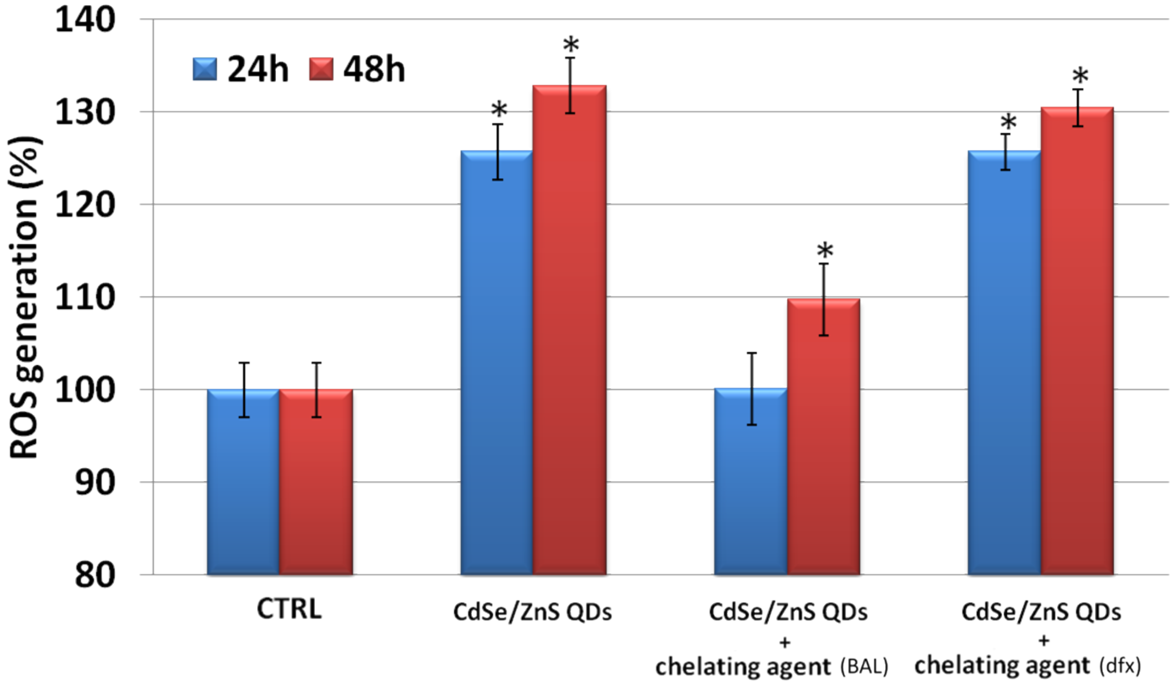

Fig. S19. Toxicity of CdSe/ZnS QDs (5 nM) in HeLa cells in absence and in presence of a specific (2,3 dithiopropanol, BAL) or unspecific (desferrioxamine, dfx) chelating agent of Cadmium ions. It is clear that, while BAL is capable to strongly reduce the toxicity of QDs, the unspecific chelator dfx is completely ineffective, showing ROS levels comparable to those elicited by the QDs alone. ROS quantification was performed via DCFH-DA assay. HeLa cells were pretreated for 30 min with/without 100 mM dfx or 1 mM BAL and then exposed to CdSe/ZnS QDs for 24/48 h. CTRL represents the negative control; values are means+s.d. Differences between treated samples and controls (n=8) were considered statistically significant for *P<0.05, non-significant for P>0.05.

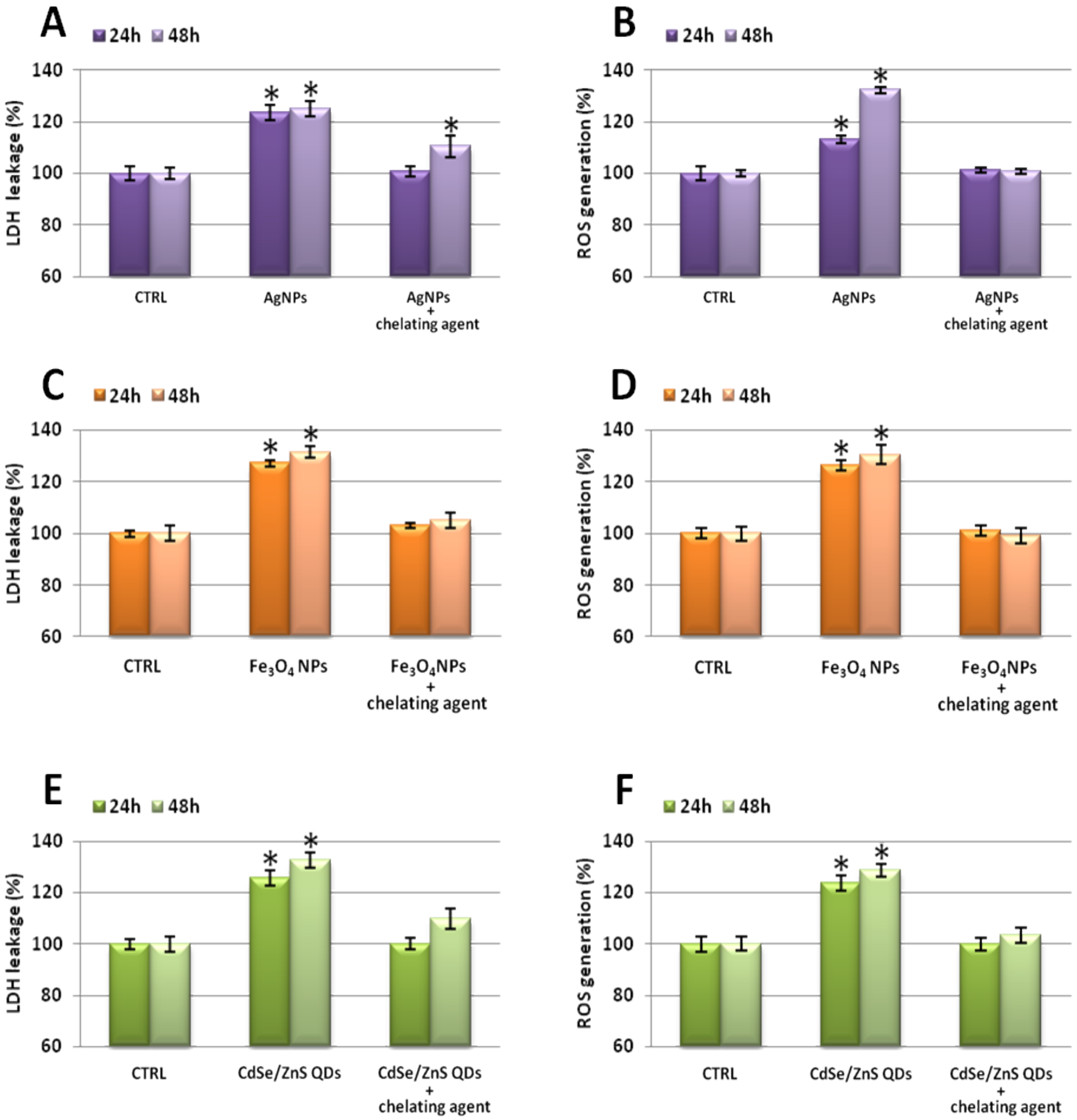

Fig. S20. Effect of specific ion chelators on membrane damage and ROS generation induced by different types of NPs on HeLa cells. Left column: Membrane integrity (LDH leakage assay); Right column: ROS quantification (DCFH-DA assay). (A,B) Treatment with AgNPs (2 nM) in presence/absence of 2,3 dithiopropanol (BAL); (C,D) Treatment with Fe_3O_4 NPs (2.5 nM) in presence/absence of desferrioxamine (dfx); (E,F) Treatment with CdSe/ZnS QDs (5 nM) in presence/absence of 2,3 dithiopropanol (BAL). The treatment with chelating agents is the same described in Fig. 5 in the main text. In all cases, the pretreatment with chelating agents suppresses almost totally the toxicity of the NPs. CTRL represents the negative control; values are means+s.d. Differences between treated samples and controls (n=8) were considered statistically significant for *P<0.05, non-significant for P>0.05.

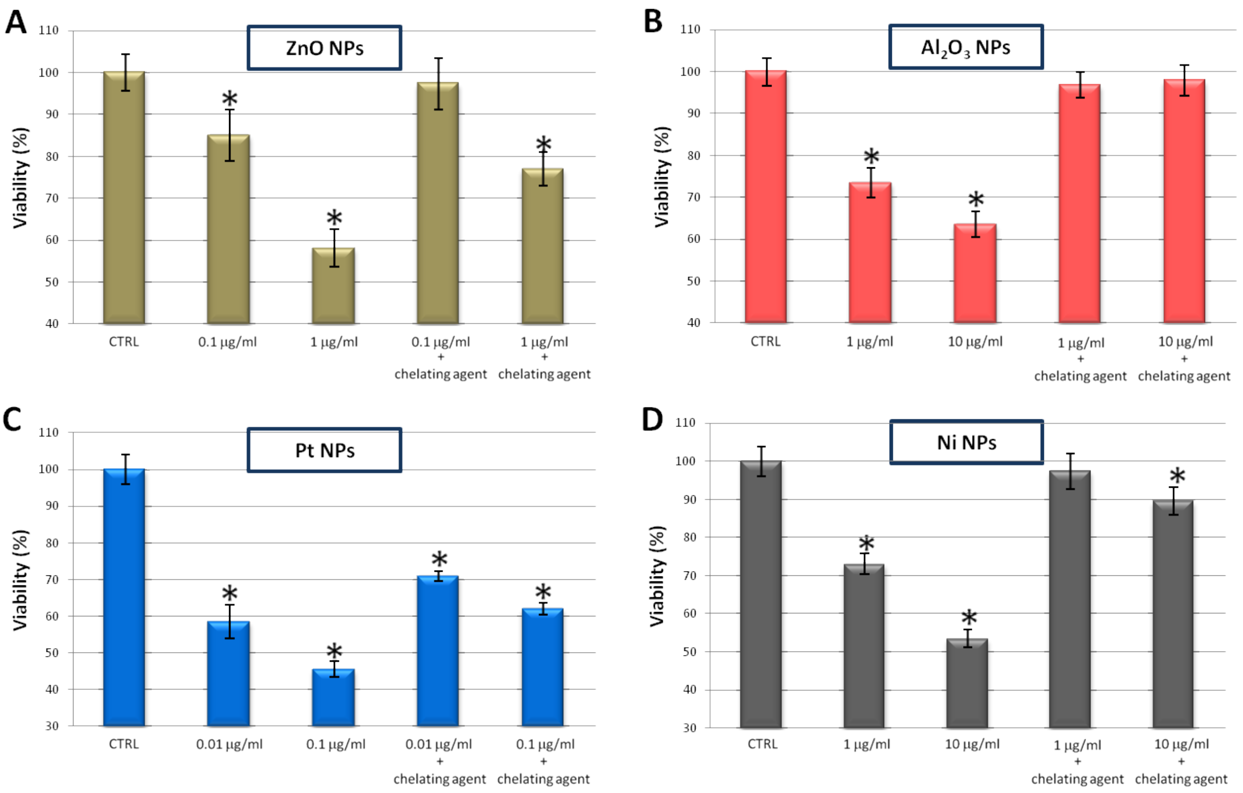

Fig. S21. WST-8 proliferation assays upon treatment with (A) zinc oxide (ZnO NPs, 24 h), (B) aluminum oxide (Al_2O_3 NPs, 48 h), (C) platinum (Pt NPs, 24h) and (D) nickel (Ni NPs, 48 h) in HeLa cells in the presence/absence of chelating agent. It is clear that the treatment with the chelating agent significantly reduced the NPs toxicity. HeLa cells were pretreated for 30 min with/without 100 µM DMSA and then exposed to the NPs for 24 or 48 h. Ctrl represents the negative control; values are means+s.d. Positive controls (not shown) were treated with 0.01% of TritonX100, displaying a strong viability decrease (ca. 80-90%) with respect to the untreated cells. CTRL represents the negative control; values are means+s.d. Differences between treated samples and controls (n=8) were considered statistically significant for *$P<0.05$, non significant for $P>0.05$.

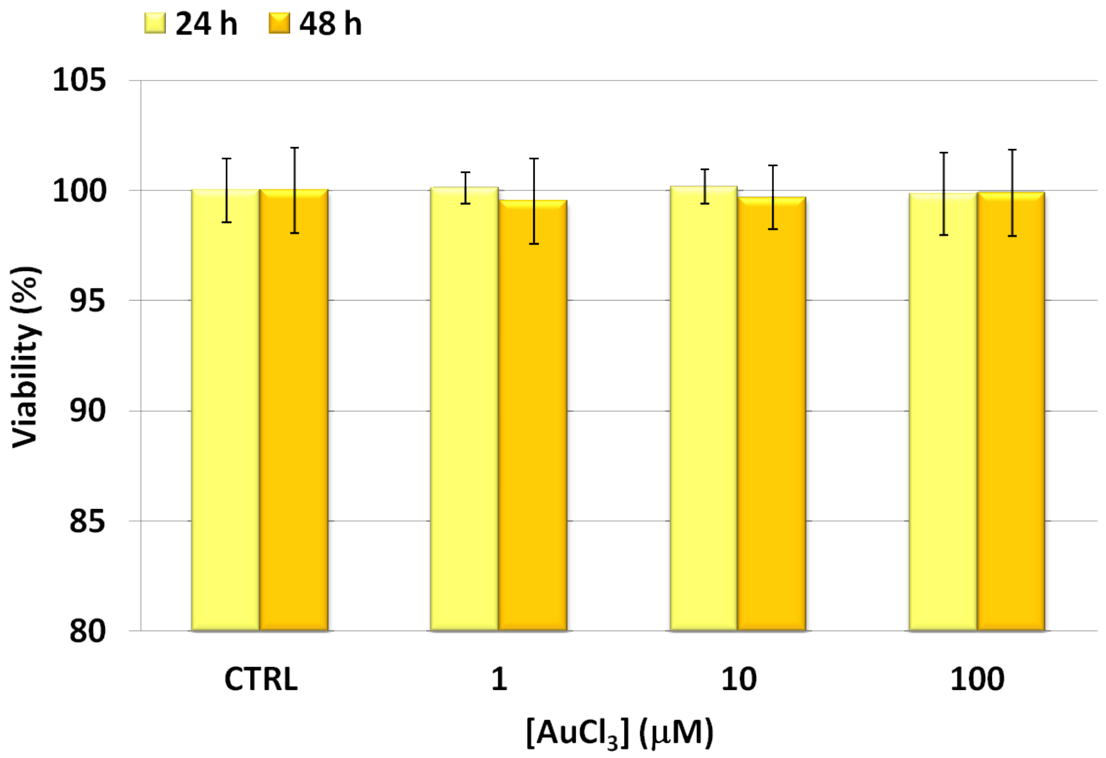

Fig. S22. Toxicity assessment of gold ions ($AuCl_3$) in HeLa cells. WST-8 proliferation assay upon treatment with increasing amount of $AuCl_3$. Note that the highest concentration of gold ions used (100 μM) corresponds to a dose of ca. 58 nM of striped and unstructured AuNPs, namely ca. 3 times higher than the maximum concentration used in all cytotoxicity assays with AuNPs. CTRL represents the negative control; values are means+s.d. and differences between treated samples and controls (n=8) were considered statistically significant for *$P<0.05$, non significant for $P>0.05$.

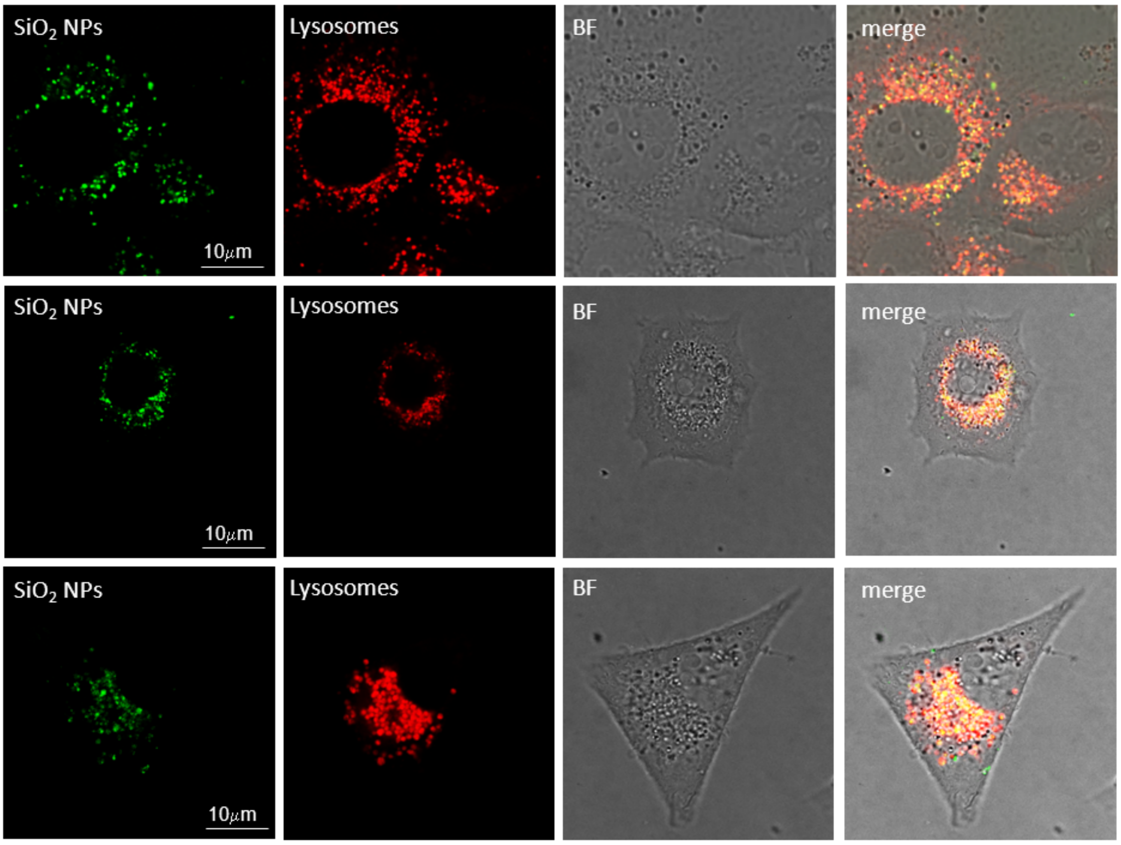

Fig. S23. Co-localization of SiO_2 NPs and lysosomes in living MCF7 cells (after 24 h incubation). Representative confocal microscopy images of fluorescent SiO_2 NPs (NPs were doped with Oregon green 488) (green channel), lysosomes (stained with LysoTracker, red channel), bright field (BF), and relative merged images. Images were taken with a Leica SP8 confocal microscope with a 40X, 1.3 NA oil immersion objective.

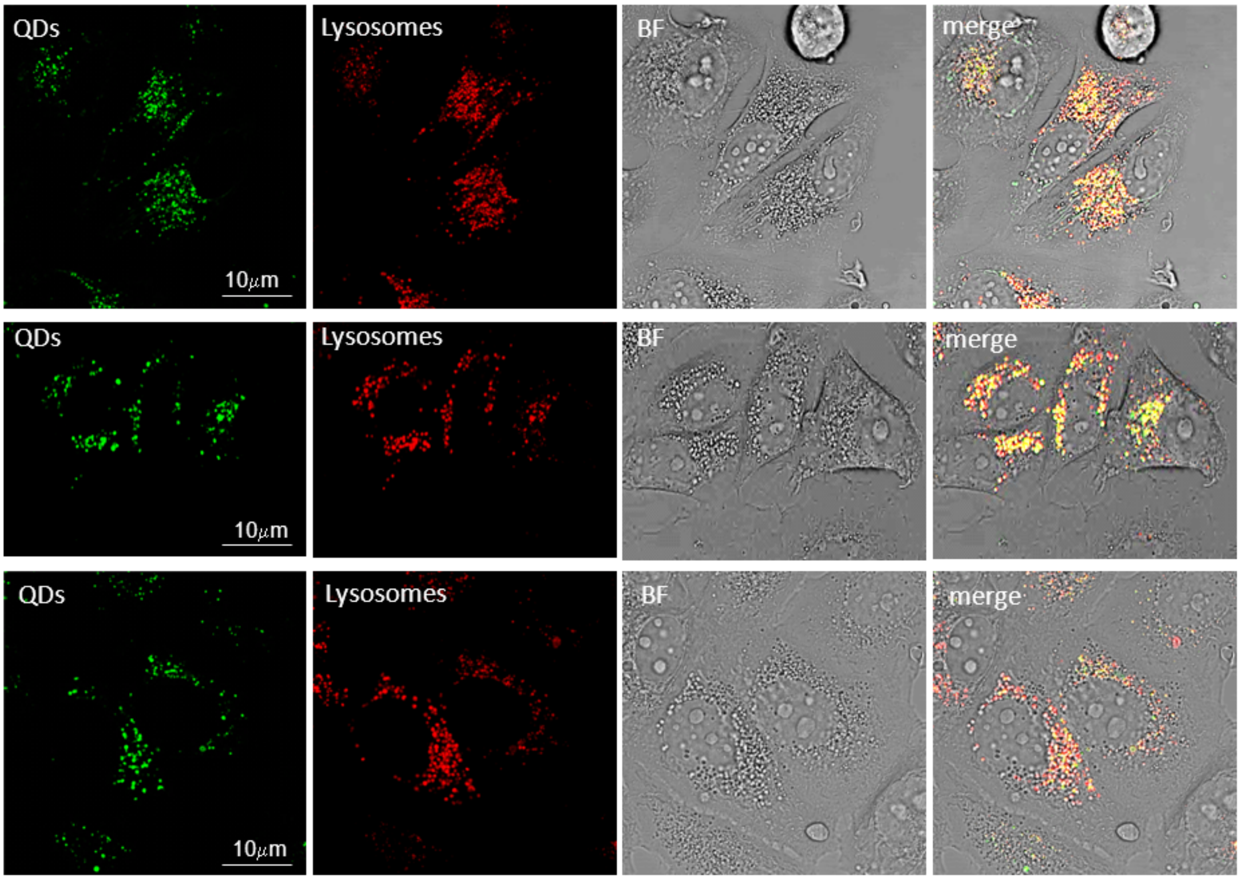

Fig. S24. Co-localization of CdSe/ZnS QDs and lysosomes in living MCF7 cells (after 24 h incubation). Representative confocal microscopy images of fluorescent QDs (green channel), lysosomes (stained with LysoTracker, red channel), bright field (BF), and relative merged images. Images were taken with a Leica SP8 confocal microscope with a 40X, 1.3 NA oil immersion objective.

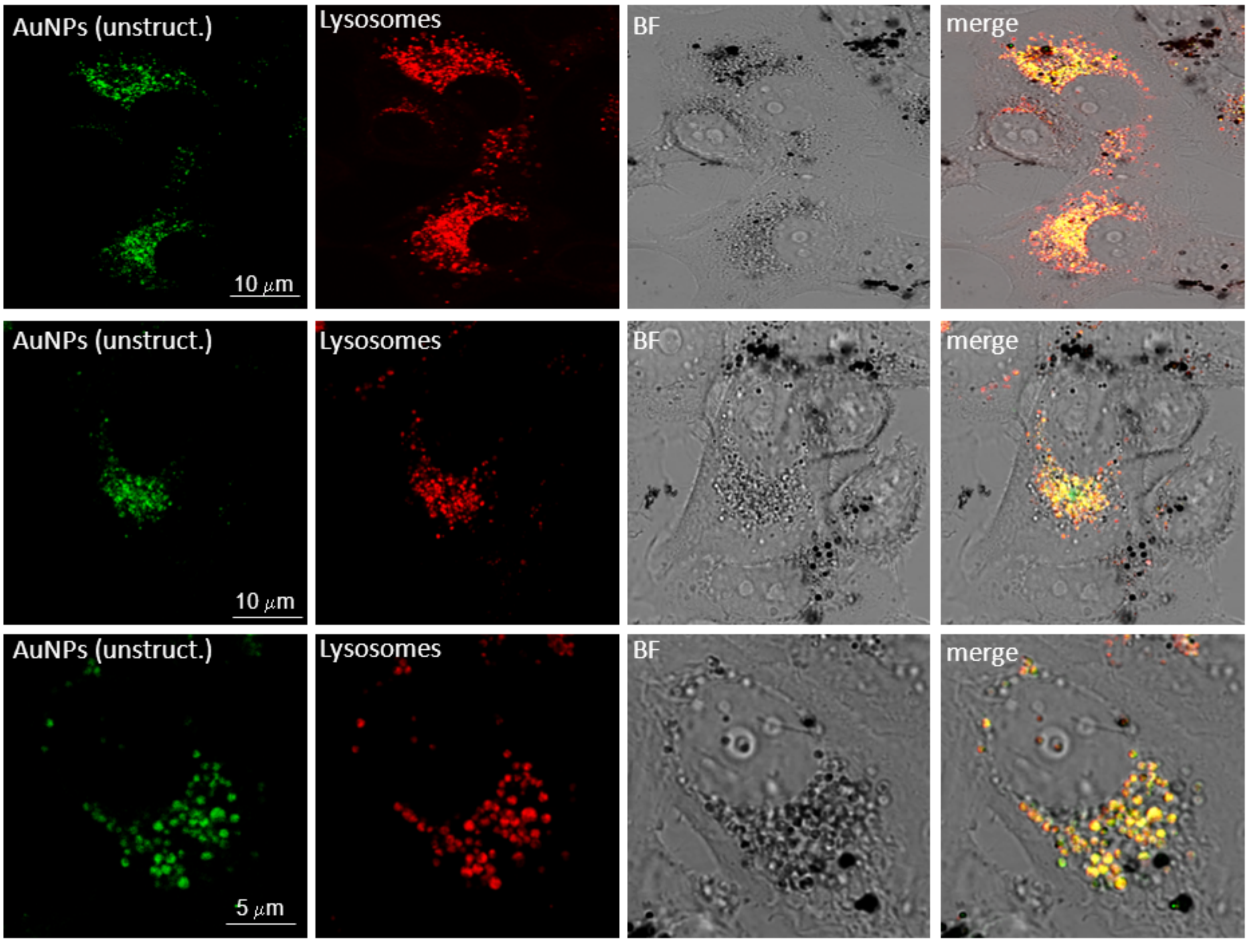

Fig. S25. Co-localization of unstructured AuNPs and lysosomes in living MCF7 cells (after 24 h incubation). Representative confocal microscopy images of fluorescent AuNPs (NPs were doped with Bodipy 650) (green channel), lysosomes (stained with LysoTracker, red channel), bright field (BF), and relative merged images. Images were taken with a Leica SP8 confocal microscope with a 40X, 1.3 NA oil immersion objective.

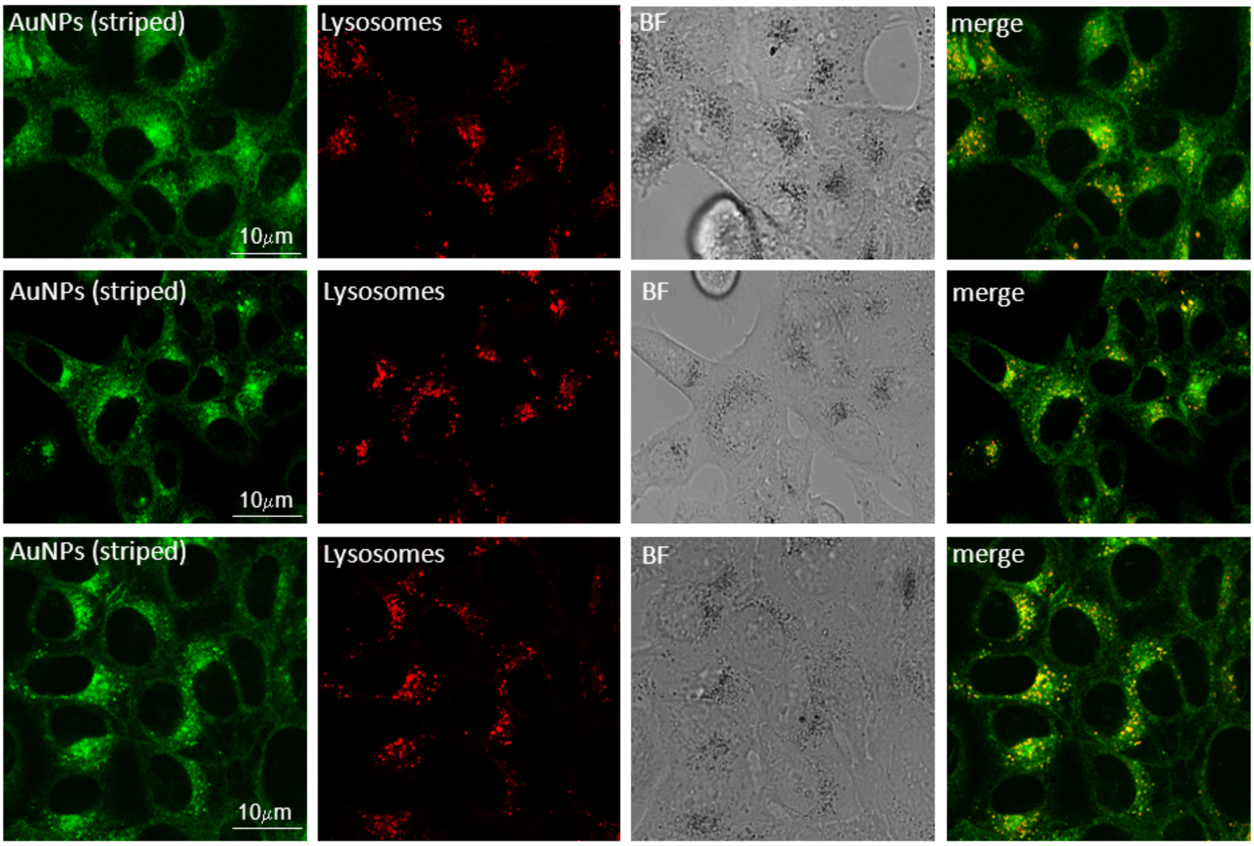

Fig. S26. Co-localization of striped AuNPs and lysosomes in living MCF7 cells (after 24 h incubation). Representative confocal microscopy images of fluorescent AuNPs (NPs were doped with Bodipy 650) (green channel), lysosomes (stained with LysoTracker, red channel), bright field (BF), and relative merged images. Images were taken with a Leica SP8 confocal microscope with a 40X, 1.3 NA oil immersion objective.

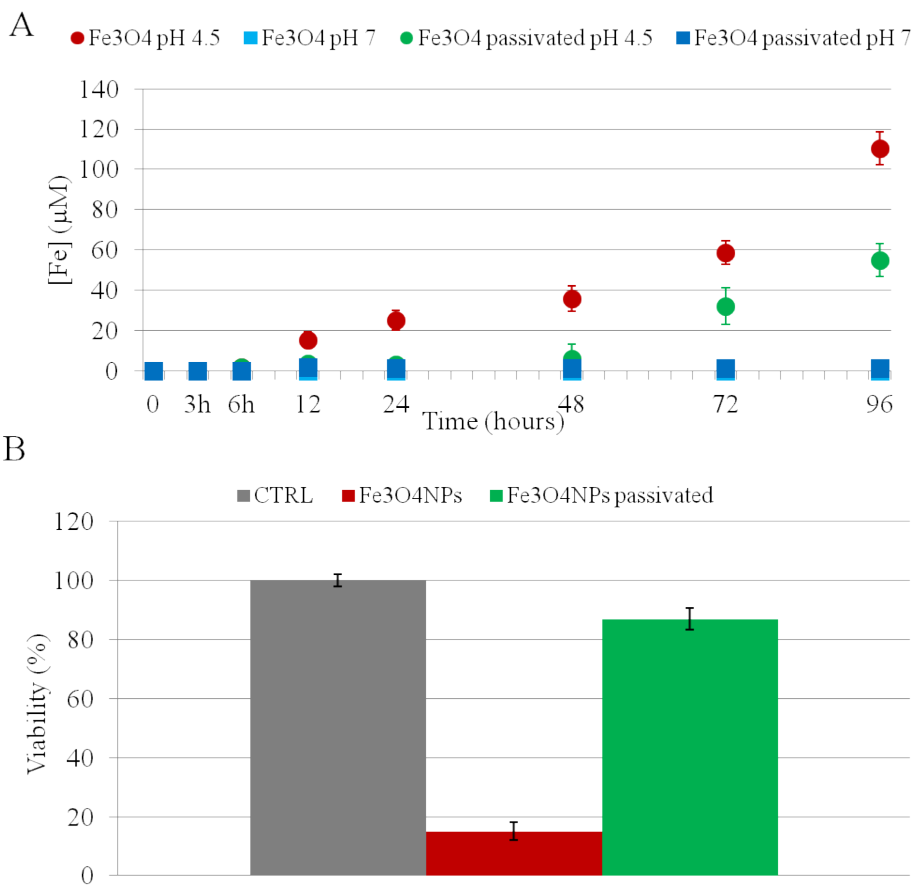

Fig. S27. Evaluation of the effects of surface passivation of Fe_3O_4 NPs on the release of iron ions and on cellular toxicity. (A) Time-dependent ion release, probed by ICP-AES, of Fe_3O_4 NPs (red circles) and passivated Fe_3O_4 NPs (green circles), 40 nM concentration, at 37 °C, at neutral (pH 7.0) or acidic (pH 4.5) conditions. (B) HeLa cells viability after 96 h treatment with 2.5 nM of Fe_3O_4 NPs (red column) and passivated Fe_3O_4 NPs (green column), evaluated through WST-8 assay. The surface of the thin silica shell surrounding the Fe_3O_4 NPs was passivated by functionalization with 3-(trihydroxysilyl)-1-propanesulfonic acid (SIT). Such surface passivation leads to a significant reduction of ion leakage from the NPs in acidic conditions (panel A), while maintaining the original characteristics of the NPs (same size and same surface charge (-28±5 mV), thus same cellular internalization); consequently, this results in a remarkable reduction of cellular toxicity (panel B). Hence, the very same NP is largely less toxic because its suitable surface engineering strongly reduces its intracellular release of toxic ions.

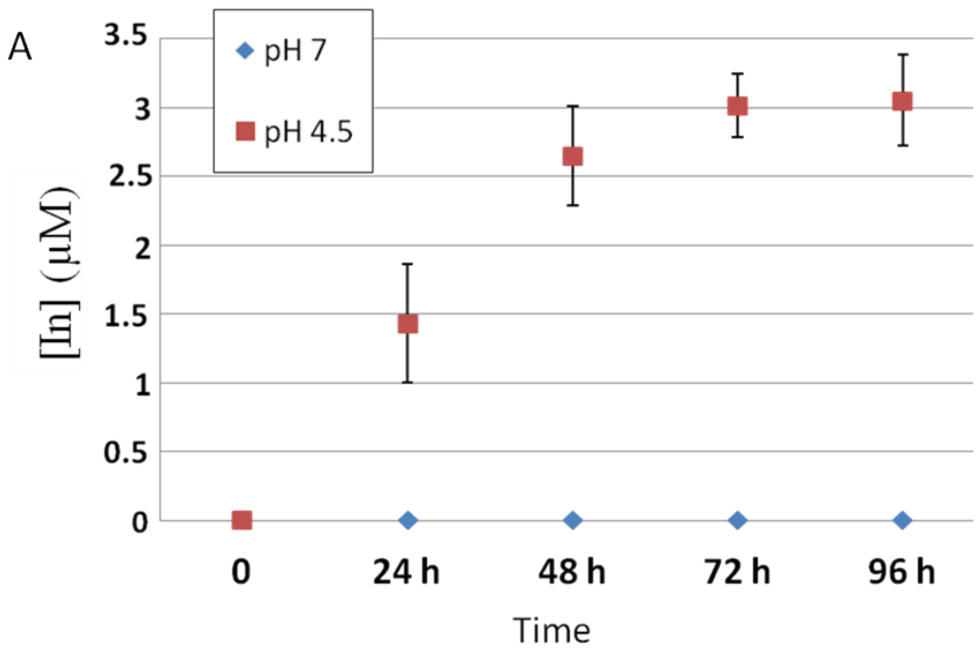

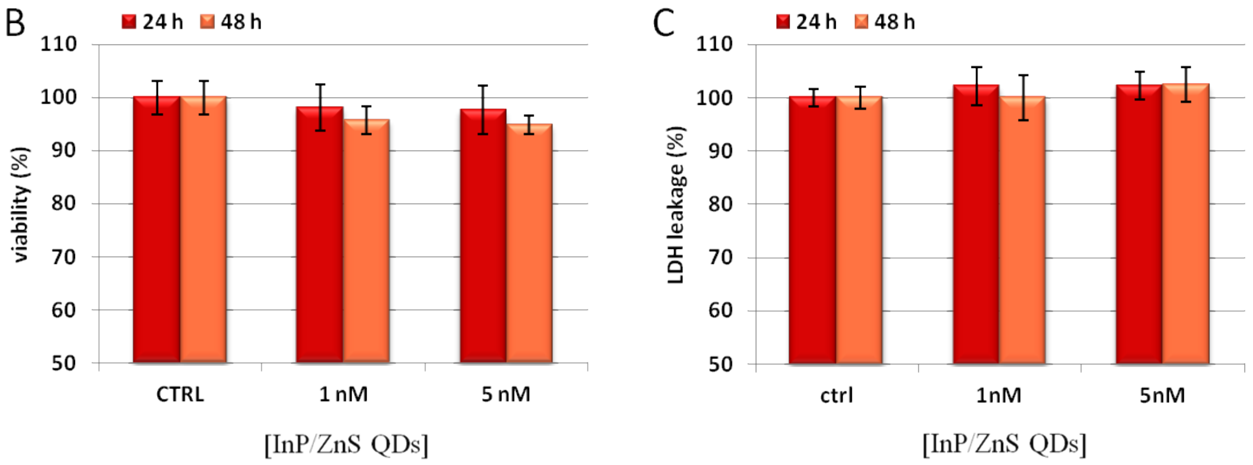

Fig. S28. (A) Time-dependent release of Indium ions from InP/ZnS QDs (5 nM), probed by ICP-AES, at 37 °C, at neutral (pH 7.0, blue symbols) or acidic (pH 4.5, red symbols) conditions. Data represent the average from 3 independent measurements (6 replicates for each experiment) and the error bars indicate the standard deviation. (B) WST-8 proliferation assay and (C) LDH assay on SH SY5Y cells incubated with two concentrations of InP/ZnS QDs at different times (24 and 48 h). Ctrl identifies the negative controls (in absence of QDs). Data are reported as mean ± SD from three independent experiments. Differences between treated samples and controls (n=8) were considered statistically non-significant for P>0.05. Although these QDs undergo degradation in acidic, lysosomal-like conditions, with release of Indium ions, it is clear that such ions are very well tolerated by the cells, which show no detectable toxicity even at high NP concentrations[20].

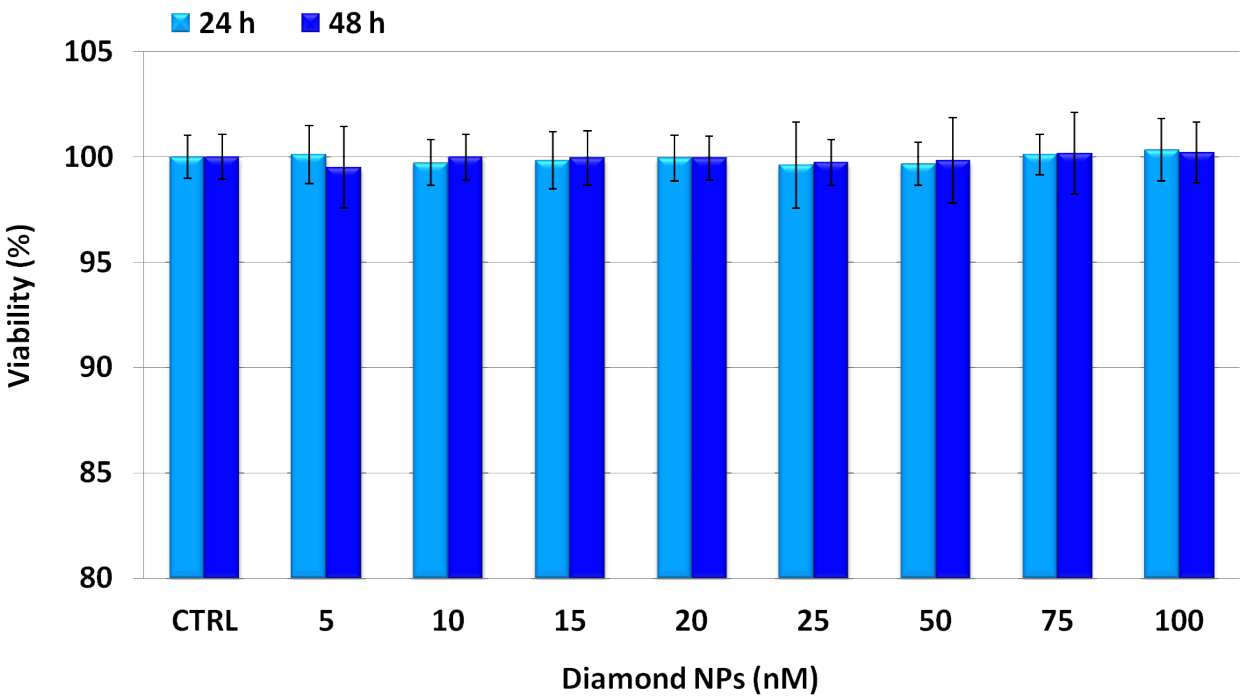

Fig. S29. Viability test of U937 cells by WST-8 assay upon treatment with nanodiamonds. Viability was monitored up to 100 nM of nanodiamonds finding no toxicity. Ctrl represents the negative control; values are means+s.d. (n=8).

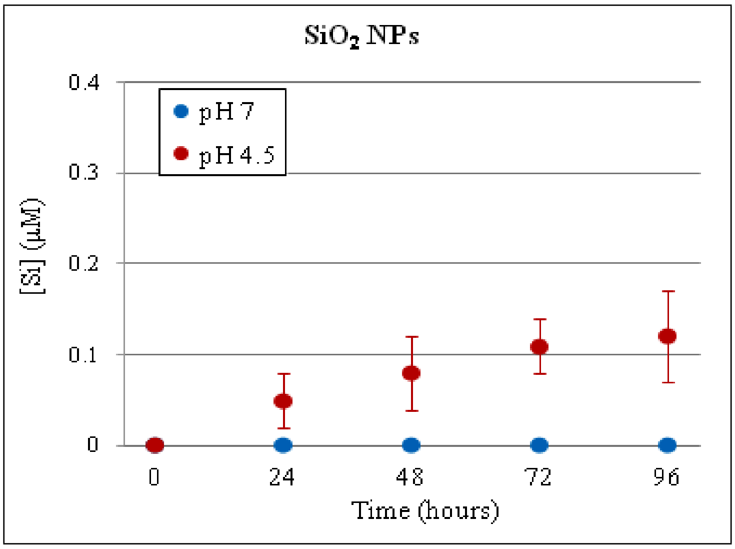

Fig. S30. Time-dependent Si release, probed by ICP-AES, of 25 nm SiO_2 NPs (40 nM concentration) at 37 °C, at neutral (blue symbols) or acidic (red symbols) conditions, according to the experimental conditions of Fig. 1. Data represent the average from 3 independent measurements (6 replicates for each experiment) and the error bars indicate the standard deviation.

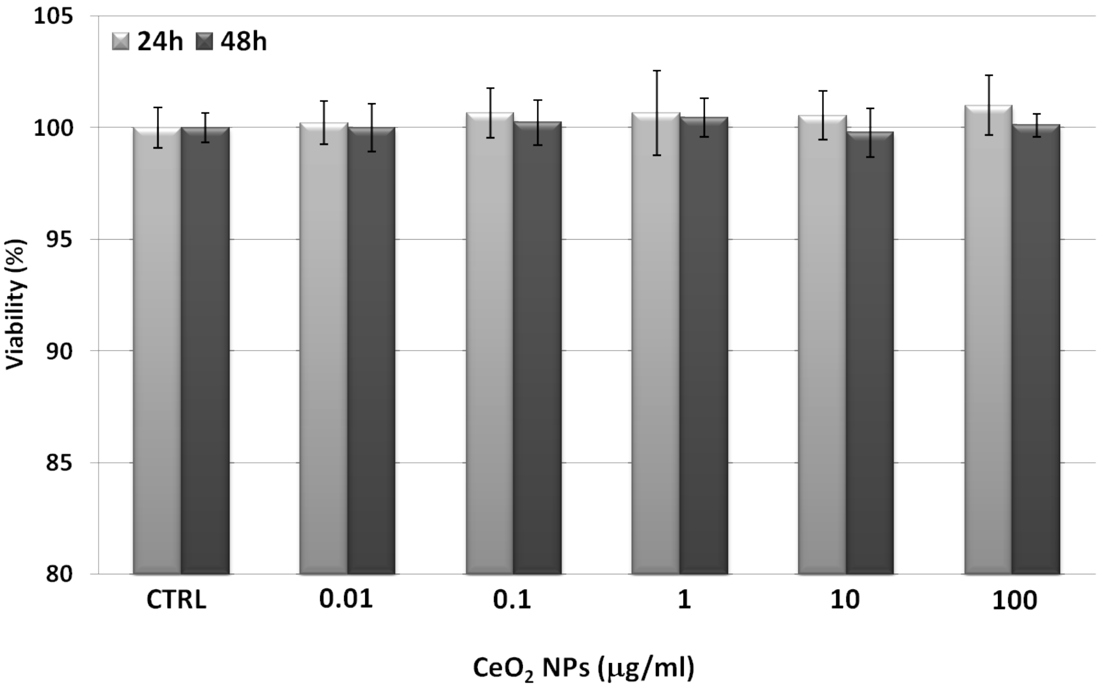

Fig. S31. Viability test of MCF7 cells by WST-8 assay upon treatment with ceria NPs. Viability was monitored up to 100 μg/ml of NPs, finding no toxicity. Ctrl represents the negative control; values are means+s.d. (n=8).

Materials and Methods

Synthesis of NPs

Striped and unstructured gold nanoparticles were prepared as previously reported[12].

Silver nanoparticles were synthesized by photochemical method by using tyrosine as a photoreducing agent. The synthesis was carried out in a laboratory reactor system fitted with UV lamp and surrounded by quartz tubing for cooling with water. The pre-cooled aqueous solution of potassium hydroxide irradiated with the UV lamp was added with tyrosine and silver ions (Ag_2SO_4) under vigorous stirring conditions. After 30 min, the AgNP suspension was warmed up to room temperature. The concentrated NP suspension was purified and separated by using sephadex G-75.

CdSe/ZnS core/shell NPs were prepared following standard colloidal synthesis procedures[13,14] and transferred from the organic phase to aqueous phase by adopting a polymer coating procedure[15].

Spherical Fe_3O_4 NPs were synthesized by a modified surfactant-assisted nonaqueous synthetic approach[16]. Iron pentacarbonyl ($Fe(CO)_5$) was decomposed in hot noncoordinating 1-octadecene solvent (ODE) in the presence of oleic acid (OLAC) as both oxygen source and capping agent at 320 °C under nitrogen atmosphere for 3 h. NPs were achieved by adjusting the concentration of the precursor (0.25 M) and the surfactant-to-precursor molar ratio (4/1). NPs were precipitated by adding 2-propanol, washed several times with acetone, and finally the precipitate was dissolved in a small amount of chloroform. The core nanoparticles were subsequently transferred into water, by coating their surface with a silica shell using an inverse microemulsion method[17]. In some specific experiments (Fig. S20), the thin silica shell of Fe_3O_4 NPs was passivated by functionalization with 3-(trihydroxysilyl)-1-propanesulfonic acid (SIT). NPs were dispersed in freshly prepared 8% (v/v) solution of SIT and the pH of the suspension was adjusted to pH=5, by adding an appropriate amount of NaOH solution (1 M); afterwards the solution was stirred overnight at 70 °C, and washed several times by centrifugation to remove the SIT excess.

SiO_2 NPs were synthesized as previously reported[18].

Nanodiamonds (NDs) were purchased from Sigma. They were functionalized with fluorescein isothiocyanate (FITC) as previously reported[19], to assess cellular internalization. Before cellular tests, NDs were purified by filtration.

ZnO, AlO, and Ni NPs were purchased from Sigma.

Pt NPs were purchased from HiQ-Nano.

InP/ZnS QDs, donated by Hicham Chibli, were synthetized according to Brunetti et al.[20]

Ceria NPs were donated by Fanny Caputo (University of Rome Tor Vergata).

Characterization of NPs

All NPs used in this study were characterized by TEM, DLS and ζ-potential.

For TEM analyses, samples were prepared by dropping a dilute solution of nanoparticles in water on carbon-coated copper grids (Formvar/Carbon 300 Mesh Cu). TEM images were recorded on a JEOL Jem1011 microscope operating at an accelerating voltage of 100 kV. The size of nanoparticles was obtained after measuring the size of more than 100 particles by TEM.

For DLS and ζ-potential measurements, a Zetasizer Nano ZS90 (Malvern, USA) equipped with a 4.0 mW He–Ne laser operating at 633 nm and an avalanche photodiode detector was used. Measurements were made at 25 °C in water (pH 7.0) or in cell culture medium, DMEM, 10% FBS. The size distributions were performed by volume (%), while the ζ-potential measurements by intensity. Each sample was measured 5 times and the results analyzed by Malvern Instruments Ltd software.

The characterization data are reported in Tab. S32

		**H_2O**		**Cell culture medium**	
**NPs type**	**TEM (nm)**	**DLS (nm)**	**ζ -potential (mV)**	**DLS (nm)**	**ζ -potential (mV)**
**Au** (unstructured)	4.3 ± 1.3	4.4 ± 1.2	-30.2 ± 1.4	32 ± 10	-22 ± 8
**Au** (striped)	4.4 ± 1.1	4.4 ± 1.1	-32.1 ± 1.2	30 ± 12	-21 ± 9
**Ag**	5.5 ± 2.0	6 ± 2.1	-25.6 ± 3.1	22 ± 2	-26 ± 9
**CdSe/ZnS**	6.0 ± 0.5	15.2 ± 1.5	-29.0 ± 3.5	37 ± 4	-18 ± 9
**Fe_3O_4**	25 ± 1.2 (10.1 ± 2.1)	27.2 ± 3.5	-31.2 ± 5.4	107 ± 13	-24 ± 4.0
**SiO_2**	24.8 ± 1.3	25.0 ± 4.0	-30.5 ± 5.0	100 ± 15.0	-21 ± 6
**NDs**	6.2 ± 3.1	68.2 ± 5.0	-44.8 ± 6.0	180 ± 10	-26 ± 5

**ZnO**	31.6 ± 5.8	52 ± 3.4	+43 ± 2.7	182 ± 17	-20 ± 2.7
**Pt**	5.7 ± 0.5	7.6 ± 1.4	-41 ± 2.3	101 ± 5	-25 ± 3
**AlO**	17.8 ± 4.3	58 ± 6	+51 ± 3.5	160 ± 29	-12 ± 6
**InP**	2.7 ± 0.7	11.3 ± 1.6	-26 ± 9	27.4 ± 1.8	-22 ± 8
**Ni**	40 ± 10.5	91 ± 16	+22 ± 3.6	156 ± 13	-21 ± 4.2
**CeO_2**	11 ± 2.6	28 ± 5	+26 ± 2	47 ± 3.6	-27 ± 3

Tab. S32. TEM, DLS and ζ-potential data of all the NPs types. The CdSe/ZnS NPs are polymer coated (see Methods), so the size detected by TEM refers to the main diameter of the CdSe/ZnS structure, while DLS data (and ζ –potential values) account for the coating. The Fe_3O_4 NPs are coated by a thin silica shell (see Methods), so the reported data refer to the final nanostructures, while the TEM value shown in brackets indicates the size of the Fe_3O_4 core (10 nm). Characterization data of the NPs are also reported in cell culture medium (DMEM supplemented with 10% FBS), namely in the same conditions used for cell experiments, after 24 h incubation. As expected, the cell culture medium elicits the formation of protein corona around the NP surface[21], changing their original size and surface charge, and causing some NP agglomeration.

Measurements of NP ion release

The evaluation of NP ion release was performed at 37 °C both in acidic conditions (sodium citrate buffer, pH 4.5, an acidic medium mimicking the lysosomal environment[22,23] and in neutral conditions (ultrapure water, pH 7 or DMEM supplemented with 10% of FBS, pH 7.4). The citrate buffer was prepared by mixing appropriate volumes of 20 mM aqueous solutions of citric acid and sodium citrate monobasic to achieve the final desired pH. Adequate volumes of stock nanoparticle suspensions were diluted in buffer and in water in order to achieve the final NP concentrations of 50 nM for AuNPs, 20 nM for CdSe/ZnS NPs, 40 nM for Fe_3O_4 NPs, 17 nM for AgNPs, and 40 nM for SiO_2 NPs. The nanoparticles were then incubated at 37 °C. The ion release was analyzed over time at 24, 48, 72 and 96 hours. At each time point, the NPs were separated from the rest of solution through filtration using Amicon filters (3000 MWCO). Filtrated solutions were collected and digested by the addition of a solution of HCl/HNO_3 3:1 (v/ v), and the amount of free ions was measured by ICP-AES (Inductively Coupled Plasma Atomic Emission Spectrometer).

Measurements of NP cellular internalization

To estimate the amount of internalized gold per cell, U937 and HeLa cells were seeded in 6 well plates (Sarsted) at a density of 10^6 cells/ml and incubated with 20 nM of striped and unstructured AuNPs for 48 h. For the inhibition studies of the endocytotic active processes, cells were pretreated for 30 min at 4 °C or at 37 °C with inhibitors: sodium azide (NaN_3, 100 µM) and 2-deoxyglucose (Dox-G, 100 µM) or Cytochalasin D (Cyt D, 10 µM) and then exposed to 20 nM of striped and unstructured AuNPs for 4 h. AuNPs untreated cells were used as control. After treatments, cells were washed twice in sterile cold PBS by centrifugation. An additional washing step using a solution of I_2/KI with etching properties was added to the cells following the procedure described by Cho et al.[24]. In particular, 1 mL of I_2/KI (0.34 mM/1.86 mM) was added to the cell well plate for 2 minutes. Afterward, cells were washed with PBS, treated with trypsin and finally centrifuged at 300 g for 5 minutes. Five replicates were analyzed for each treatment (3 independent experiments). Then, samples were digested and analyzed by ICP as described above.

Measurements of intracellular NP ions

To estimate the amount of intracellular NP ions, HeLa cells were seeded in 150 cm^2 flasks (Sarsted) at a density of 10^6 cells/ml and exposed to 20 nM of striped and unstructured AuNPs for 48 h. After that, cells (5 x 10^7) were collected and washed with cold PBS by centrifugation. Each

sample was then lysed and filtered by Amicon Ultra-4 (3000 MWCO) following the manufacturer's instructions. The obtained filtrated solutions were finally analyzed by ICP-AES for the determination of gold ions. Untreated cells were used as control. Five replicates were analyzed for each treatment (3 independent experiments).

Cell cultures

Human cervix carcinoma cells, HeLa (ICLC number: HTL950239), human lymphoma cells, U937 (ICLC number: HTL94002), MCF7 human breast adenocarcarcinoma cells (ICLC number: HTL95021), SH SY5Y human neuroblastoma cells (ICLC number: HTL95013), Caco-2 human colon adenocarcinoma cells (ICLC number: HTL97023), were purchased from Interlab Cell Line Collection, IST (Genova, Italy). Huh7 human hepatoma were purchased from American Tissue Type Culture Collection (LGC Standards S.r.l., Milano, Italy). All cell lines were cultured in 75 cm^2 flasks (Sarstedt) in high glucose DMEM, supplemented with 10% (v/v) Fetal Bovine Serum (Sigma Aldrich), 1% (v/v) 10000 U/ml Penicillin and 10000 U/ml Streptomycin (Sigma Aldrich) 2mM L-Glutamine (Sigma Aldrich) + 1% non-essential amino acids (Sigma Aldrich). U937 were cultured in RPMI 1640 media (Sigma Aldrich) supplemented with 10% (v/v) Fetal Bovine Serum, 1% (v/v) 10000 U/ml Penicillin and 10000 U/ml Streptomycin and 2mM L-Glutamine. For all the experiments, cells were maintained under standard cell culture conditions (5% CO_2, 95% humidity and 37 °C) and harvested every 3 days.

WST-8 Cytotoxicity Assay

The metabolic activity of all the aforementioned cells was measured after 24 and 48 h of exposure to striped and unstructured AuNPs, using a standard WST-8 assay (Cell Counting Kit-8, code: 96992, Sigma) and following the experimental procedures described by Sabella et al.[25]. Briefly, cells were treated with increasing concentration of AuNPs ranging from 0.03, 0.1, 0.3, 1, 3, 4, 5 μg/mL (corresponding to 0.1, 0.5, 1, 5, 10, 15, 20 nM). TritonX100 (Sigma) (at a final concentration of 0,01%) was employed as positive control, leading to a cellular viability reduction in the range of 80-90% with respect to the negative control (data not shown in the graphs). After reacting each well with aliquots of 10 μL of WST-8 solution for 3 h, the orange WST-8 formazan product was measured by using a microplate reader (Fluo Star Optima, BMG LABTECH) at the selected wavelength (460 nm). Data were analyzed by MARS Data Analysis Software (BMG LABTECH) and cytotoxicity expressed by using the previously reported equation[25]. Data were

indicated as mean ± SD for 4 independent experiments (8 replicates for each experiment). The same procedure was applied for all other NPs at the working concentration ranges.

Experiments with AuNP conjugates: Striped AuNPs (200 nM) in aqueous solution were mixed with 4-fold excess of proteins (Transferrin or Apolipoprotein B-100, Sigma) in a glass vial. These solutions were allowed to mix *o.n.* at room temperature while stirring. The resulting solutions containing the conjugates (Tstriped or Astriped) were purified by repeated centrifugation steps[26]. The AuNP conjugates were characterized by UV-vis, TEM and DLS, showing no detectable aggregation and exhibiting an average hydrodynamic diameter of 11±6 nm and 13±5 nm for transferrin and apolipoprotein, respectively. Chemical-physical characteristics of the conjugates are reported below.

		**H_2O**	
**NP type**	**TEM (nm)**	**DLS (nm)**	**ζ -potential (mV)**
**Tstriped)**	4.4 ± 1.1	11 ± 6	-12 ± 5
**Astriped**	4.4 ± 1.1	13 ± 5	-15 ± 4

Table S33: TEM, DLS and ζ-potential data of Tstriped and Astriped AuNPs.

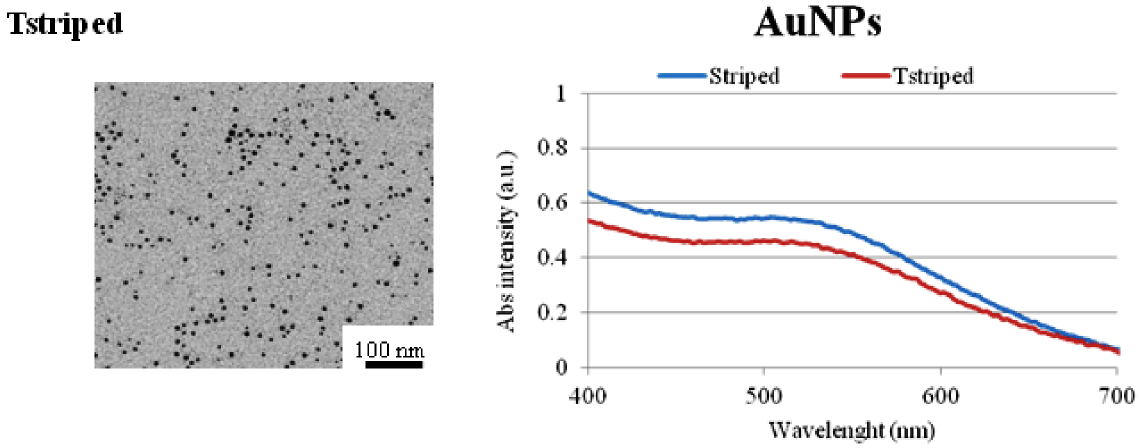

Fig. S34. Representative TEM image of Tstriped AuNPs. UV-vis spectrum of Tstriped AuNPs as compared to that of striped AuNPs. UV-vis curve evidences the absence of agglomeration/aggregation in solution containing the Tstriped conjugate.

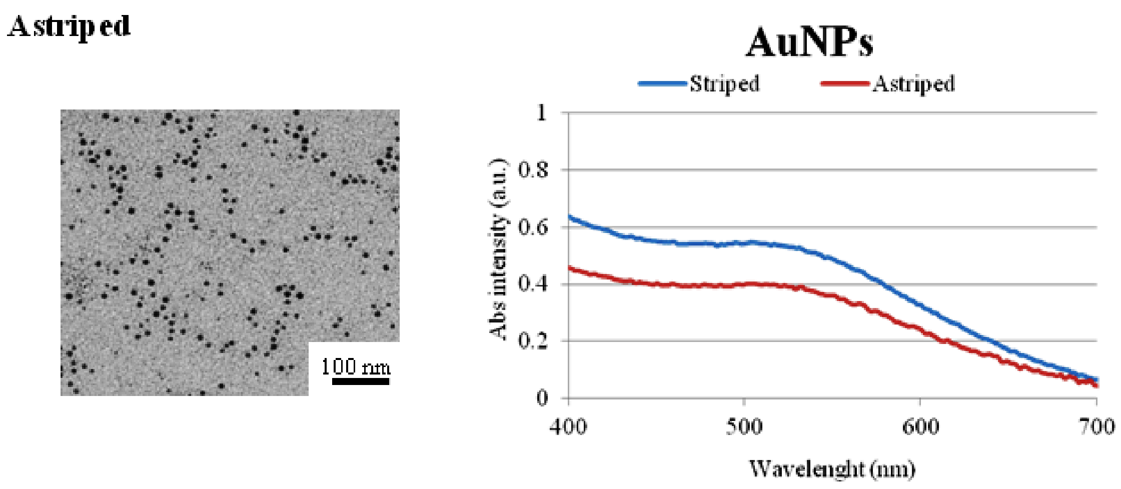

Fig. S35. Representative TEM image of Astriped AuNPs. UV-VIS spectrum of Astriped AuNPs as compared to that of striped AuNPs. UV-VIS curve evidences the absence of agglomeration/aggregation in solution containing the Astriped conjugate.

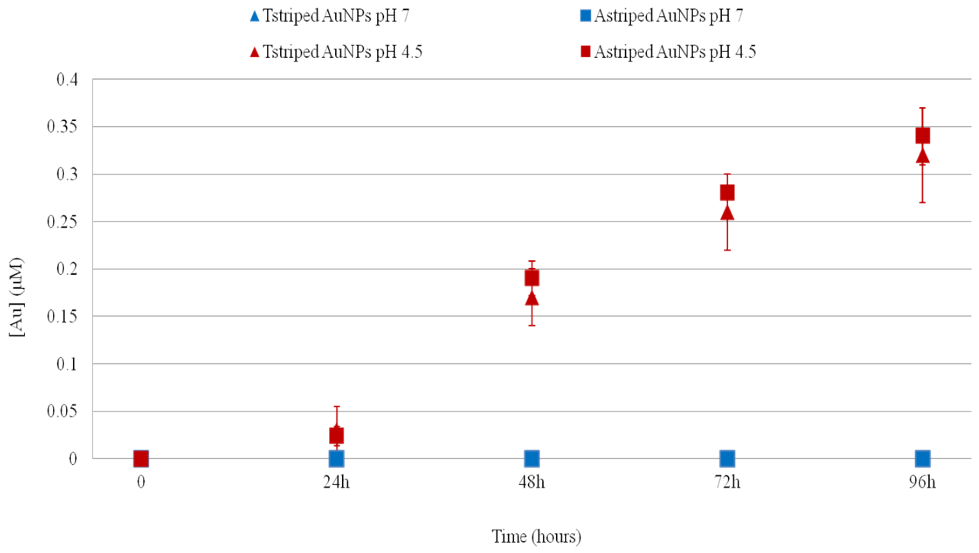

Fig. S36. Time-dependent gold ion release from Tstriped and Astriped AuNPs (50 nM), probed by ICP-AES, at 37 °C, at neutral (pH 7.0, blue symbols) or acidic (pH 4.5, red symbols) conditions. Data represent the average from 6 independent measurements and the error bars indicate the standard deviation. As shown, the two AuNP bioconjugates release comparable amounts of gold ions in acidic conditions, showing the same behavior of bare AuNPs.

Cells were exposed to unstructured, Tstriped or Astriped AuNPs at a concentration of 65 nM for 12 h in serum free conditions, afterwards the medium was replaced with fresh, fully supplemented medium without NPs, and WST-8 viability measurements were done at 24 and 48 h as described above.

WST-8 Cytotoxicity Assay in the presence of lysosomotropic and chelating agents

To prove the direct role of intracellular NP ions (whose release is enhanced by acidic lysosomal conditions) in inducing toxicity, cells viability was monitored by WST-8 proliferation assay in the presence of lysosomotropic agents enabling the increase of the acidic lysosomal pH[27]. 24 h after seeding, HeLa cells were pretreated for 30 min with chloroquine (5 μM) (Sigma) or ammonium chloride (5 mM) (Sigma) and then exposed to 2.5 nM of Fe_3O_4 NPs or 20 nM of unstructured AuNPs for 48h. The WST-8 proliferation assay was carried out using 4 independent experiments (8 replicates for each experiment) as described above.

As a further demonstration of the direct relationship between the intracellular released ions by NPs and viability reduction, cells viability was monitored by WST-8 proliferation assay (as well as by ROS and LDH assay) in the presence of metals chelating agents, such as 2,3 dithiopropanol (BAL)[28] and the iron chelator, desferrioxamine (dfx)[29]. 24 h after seeding, HeLa cells were pretreated for 30 min with BAL (1 μM) and then exposed to NPs (20 nM of striped or unstructured AuNPs, 2 nM of AgNPs, 5 nM of CdSe/ZnS QDs) for 24-48 h. In the case of Fe_3O_4 NPs, HeLa cells were pretreated for 30 min with dfx (100 μM) and exposed to 2.5 nM of the iron NPs for 24/48 h. The WST-8 proliferation assay was carried out using 4 independent experiments (8 replicates) as described above. Untreated cells were used as control, while cells pretreated with the lysosomotropic agents or chelating agents without further NP exposure were tested for the toxicity of the applied chemicals (finding no toxicity for both the chemicals used, data not shown).

The same procedure was applied for other NPs (Fig. S21) at the working concentration ranges but with the use of DMSA (100 μM).

WST-8 Cytotoxicity Assay in the presence of $AuCl_3$

Cellular toxicity was assessed in human HeLa upon treatment with increasing concentrations (1, 10 and 100 μM) of a gold salt, $AuCl_3$. Freshly prepared stock solution of gold salt in water was added to cell culture medium and to cells at the desired concentrations for 24/48 hours. The WST-8 proliferation assay was carried out using 4 independent experiments (8 replicates) as described above.

ROS quantification by DCFH-DA assay

Oxidative stress of all the aforementioned cells was determined after 24 and 48 h of exposure to striped and unstructured AuNPs following the DCFH-DA assay procedures as previously reported[25]. Cells were treated at the same final concentrations of striped and unstructured AuNPs employed for the aforementioned viability assay. In this case, as positive control, a free radical generator (H_2O_2 at a concentration of 100 μM) was used, finding that it caused an increase of ROS around 190-220% with respect to the untreated cells (data not shown in the graphs). The fluorescence generated by cells from each well was measured immediately, and after 30 min of reaction with DCFH-DA by using Fluo Star Optima. Fluorescence quantification and data analyses were carried out as reported elsewhere[25]. Data were reported as mean ± SD for 4 independent experiments (8 replicates).

The same procedure was applied for all other NPs at their working concentration ranges.

Caspase-3 activation

Caspase-3 activity was detected by using the Caspase 3 Colorimetric Assay Kit (CASP-3-C Sigma) and experiments were carried out according to the manufacturer's recommended procedures. All employed cells (1×10^7) were collected after 24 and 48 h of incubation in the presence of striped and unstructured AuNPs at concentration values ranging from 0.1 to 20 nM. After treatments, cells were lysed on ice in the presence of 50 mL of lysis buffer for 10 min. Afterwards, 50 mL of reaction buffer containing dithiothreitol (DTT) and 5 mL of caspase-3 substrate solution (acetyl-Asp-Glu-Val-Asp p-nitroanilide [Ac-DEVD-pNA] were added. The concentration of the released pNA from the substrate is calculated by measuring absorbance at 405 nm by using Fluo Star Optima. Data were collected by Control Software and elaborated with MARS Data Analysis Software (BMG LABTECH). The caspase 3 activity of each sample was expressed in μmol pNA released per min per ml of cell lysate; the percentage of caspase 3 activity was calculated using the following equation: $(\text{Activity}_{\text{treated sample}}/\text{Activity}_{\text{control}}) \times 100$. Data were expressed as mean ± SD. The reproducibility of the results was guaranteed by performing four independent experiments (8 replicates).

Membrane damage by LDH assay

In these experiments, cells were seeded in a 96-well plate and treated with NPs, following the procedures previously reported.[20] After 24 and 48 hours of cells-NPs interaction, the lactate

dehydrogenase (LDH) leakage assay was performed in microplates by applying the CytoTox-ONE Homogeneous Membrane Integrity Assay reagent (Promega), following manufacturer's instructions. LDH released in the extracellular environment was measured in a Fluo Star Optima (BMG LABTECH) microplate reader. The same assay was performed onto untreated cells as negative control. Results were normalized with respect to the negative control (expressed as 100%). The positive control consisted in treatment of the cells with 0.9% Triton X-100 and gave leakage values in the range of 700-800% (data not reported). Data were expressed as mean ± SD.

Thioredoxin reductase (TrxR) activity

<u>Thioredoxin reductase (TrxR) inhibition:</u> The assay was performed by using Thioredoxin reductase assay kit (code: CS0170, Sigma) following the manufacturer's instructions. Briefly, in a 96-wells microplate, aliquots of rat liver thioredoxin at a final concentration of 0.5 µg/ml were incubated at 25 °C in 200 µL of working buffer containing 1 mM K-phosphate (pH 7.4), 10 mM EDTA, 0.24 mM NADPH and 10 µL of filtered solutions containing the released ions from striped and unstructured AuNPs incubated at the different pH (4.5 or 7.4) for 96 h (see Fig. 1). Kinetic reactions were started by the addition to all samples and reference of 3 mM of DTNB [5,50-dithiobis (2-nitrobenzoic acid)] and its reduction was followed spectrophotometrically at 405 nm for 30 min. Enzyme activity was calculated taking into account that 1 mole of NADPH yields 2 moles of CNTP anion (reduced DTNB)[30]. Enzyme activity was expressed in µmol/min/ml and measured by MARS Data Analysis Software (BMG LABTECH). Specific enzyme activity of the non-inhibited reaction (CTRL) was 0.05 µmol/min/ml. As positive control, we used a solution of inhibitor (Auranofin, 1 µM). The percentage of TrxR activity was calculated using the following equation: $(\text{Activity}_{\text{treated sample}}/\text{Activity}_{\text{control}}) \times 100$. Data were expressed as mean ± SD. The reproducibility of the results was guaranteed by performing four independent experiments (8 replicates).

<u>TrxR inhibition by AuNP treatments in HeLa and U937 cells</u>: To directly prove the inhibition of cellular TrxRs, HeLa cells and U937 (1×10^8) were grown in 10 cm petri dishes at sub-confluence and treated with striped and unstructured AuNPs (15 nM) or 5 nm citrate capped AuNPs (15 nM), and Auranofin (1 µM, Sigma), for 48 h of incubation. After that, cells were collected, washed with cold PBS and centrifuged. Each sample was then lysed with CelLytic M, Mammalian Cell Lysis/Extraction Reagent (Sigma), and immediately before the use, Protease Inhibitor Cocktail (Sigma) was added. After centrifugation (15,000 g), aliquots of extracted proteins were quantified by Bradford assay and utilized for the evaluation of the activity of the contained TrxR as described

above. Results are reported as percentage of TrxR activity with respect to the control and plotted versus the respective treatments. Data were expressed as mean ± SD. The reproducibility of the results was guaranteed by performing four independent experiments (8 replicates).

In vivo experiments

The flies of wild-type *Drosophila melanogaster* (Oregon R+) were cultured at 24±1 °C on standard *Drosophila* food, containing agar, corn meal, sugar, yeast and nepagin (methyl-p-hydroxybenzoate). Striped and unstructured AuNPs were formulated in the *Drosophila* diet (dose: 0.36 μg/g per day), by dispersing the particles in the food. In particular, the NP solution was added to the food before solidification, mixed strongly and finally poured into vials.

For lifespan studies, newly eclosed flies were collected and housed at a density of 10 males and 10 females, separately, per each vial. At least 10 vials were used per treatment (total of 100 males and 100 female flies per lifespan) for a total number of 200 flies for each treatment. Flies were transferred into fresh food every 3–4 days, and dead flies were counted every day until all died.

Table S37. Summary of the experiments performed in this work and relative findings.

Objectives (or concepts)	Experiments	Results (Figures/Tables)
Physical-chemical characterization of NPs in water and in culture medium	• TEM, DLS, ζ -potential, UV-vis	• Tab. S32, Tab. S33, Figs. S34-35
Lysosomal-like environment is capable of promoting degradation/corrosion of metal containing NPs	• Time-dependent ion release of different NPs using conditions that mimic either lysosomal or cytoplasmic environments (*i.e.,* neutral pH, both in water and biological medium, or acidic conditions).	• Fig. 1; Figs. S5, S27A, S28A, S30, S36.<br><br>Note: Figs. S1-S4 report more detailed analyses of NPs degradation in acidic environment
The NPs entrance mechanism in cells (and consequently their intracellular localization) dictates their toxicity (lysosomal conditions promote NPs degradation and release of metal ions, unlike cytosolic environment)	• Evaluation of toxicity (viability, ROS, and caspase assays) of AuNPs with different cellular entrance mechanisms (6 cell lines used)<br>o Uptake studies of striped and unstructured AuNPs<br>o Co-localization studies of striped and unstructured AuNPs<br>o Evaluation of toxicity using T-striped and A-striped AuNPs<br>o In vivo toxicity studies with striped and unstructured AuNPs in *Drosophila melanogaster*<br><br>• Measurement of ion cargoes released intracellularly by the striped and unstructured AuNPs	• Fig. 2 and Figs. S8-S12<br>o Figs. S6 and S7<br>o Figs. S25 and S26<br>o Fig. S13, Tab. S33, Figs. S34-36<br>o Fig. S14<br><br>• Fig. 4

The intracellular toxicity of metal-containing NPs is mainly due to their intracellular (lysosomal) release of ions	• Evaluation of toxicity (viability, ROS, LDH assays) of metal containing NPs in the presence/absence of chelants<br>o Uptake studies of NPs in the presence of chelants<br>o Evaluation of spurious off-target effects of chelants<br>• Evaluation of toxicity of gold salts<br>• Evaluation of toxicity of metal containing NPs in the presence/absence of lysosomotropic agents	• Fig. 5, Figs. S20, S21<br>o Fig. S18<br>o Fig. S19<br>• Fig. S22<br>• Fig. 6
Demonstration that toxicity of AuNPs follows the same molecular mechanisms activated by gold ions, namely inhibition of the TrxR enzyme, leading to mitochondrial membrane depolarization and/or inactivation of mitochondrial enzymes	• Evaluation of TrxR activity in cells after treatment with striped and unstructured AuNPs (two cell lines)<br>o Cells viability assay<br>o Evaluation of activity of extracted TrxR by the ions released by AuNPs in same conditions of Fig.1<br>o Evaluation of TrxR activity in cells after treatment with citrate capped AuNPs	• Fig. 3B, S16<br>o Fig. S15<br>o Fig. 3A<br>o Fig. S17
Description of a general mechanism for intracellular toxicity of metal-containing NPs (LETH mechanism)	• Schematic of LETH mechanisms and co-localization studies	• Fig. 7, Figs. S23-26
Application of LETH model to identify by design biocompatible nanoparticles	• Toxicity studies of bare and passivated Fe_3O_4 NPs<br>• Toxicity of CdSe/ZnS QDs *vs*. InP/ZnS QDs<br>• Toxicity of ND NPs	• Fig. S27<br>• Fig. S28<br>• Fig. S29

	• Toxicity of silica NPs<br>• Toxicity of ceria NPs	• Fig. S30<br>• Fig. S31

References

1. Liu, J. & Hurt, R.H. Ion release kinetics and particle persistence in aqueous nano-silver colloids. *Environ Sci Technol* **44**, 2169-2175 (2010).
2. Shaw, I.C. Gold-based therapeutic agents. *Chem Rev* **99**, 2589-2600 (1999).
3. Ott, I. On the medicinal chemistry of gold complexes as anticancer drugs. *Coordination Chemistry Reviews* **253**, 1670-1681 (2009).
4. Zhong, L., Arner, E.S. & Holmgren, A. Structure and mechanism of mammalian thioredoxin reductase: the active site is a redox-active selenolthiol/selenenylsulfide formed from the conserved cysteine-selenocysteine sequence. *Proc Natl Acad Sci U S A* **97**, 5854-5859 (2000).
5. Gromer, S., Arscott, L.D., Williams, C.H., Jr., Schirmer, R.H. & Becker, K. Human placenta thioredoxin reductase. Isolation of the selenoenzyme, steady state kinetics, and inhibition by therapeutic gold compounds. *J Biol Chem* **273**, 20096-20101 (1998).
6. Mustacich, D. & Powis, G. Thioredoxin reductase. *Biochem J* **346 Pt 1**, 1-8 (2000).
7. Barnard, P.J. & Berners-Price, S.J. Targeting the mitochondrial cell death pathway with gold compounds. *Coordination Chemistry Reviews* **251**, 1889-1902 (2007).
8. Rigobello, M.P.*, et al.* Gold complexes inhibit mitochondrial thioredoxin reductase: consequences on mitochondrial functions. *J Inorg Biochem* **98**, 1634-1641 (2004).
9. Porter, A.G. & Janicke, R.U. Emerging roles of caspase-3 in apoptosis. *Cell Death Differ* **6**, 99-104 (1999).
10. Tait, S.W.G. & Green, D.R. Mitochondria and cell death: outer membrane permeabilization and beyond. *Nat Rev Mol Cell Biol* **11**, 621-632 (2010).
11. Marzano, C.*, et al.* Inhibition of thioredoxin reductase by auranofin induces apoptosis in cisplatin-resistant human ovarian cancer cells. *Free Radic Biol Med* **42**, 872-881 (2007).
12. Verma, A.*, et al.* Surface-structure-regulated cell-membrane penetration by monolayer-protected nanoparticles. *Nat Mater* **7**, 588-595 (2008).
13. Dabbousi, B.O.*, et al.* (CdSe)ZnS core-shell quantum dots: Synthesis and characterization of a size series of highly luminescent nanocrystallites. *J Phys Chem B* **101**, 9463-9475 (1997).
14. Peng, Z.A. & Peng, X.G. Formation of high-quality CdTe, CdSe, and CdS nanocrystals using CdO as precursor. *J Am Chem Soc* **123**, 183-184 (2001).
15. Pellegrino, T.*, et al.* Hydrophobic nanocrystals coated with an amphiphilic polymer shell: A general route to water soluble nanocrystals. *Nano Lett* **4**, 703-707 (2004).
16. Hyeon, T., Lee, S.S., Park, J., Chung, Y. & Bin Na, H. Synthesis of highly crystalline and monodisperse maghemite nanocrystallites without a size-selection process. *J Am Chem Soc* **123**, 12798-12801 (2001).
17. Vogt, C.*, et al.* High quality and tuneable silica shell–magnetic core nanoparticles. *J Nanopart Res* **12**, 1137-1147 (2010).

18. Malvindi, M.A., *et al.* SiO2 nanoparticles biocompatibility and their potential for gene delivery and silencing. *Nanoscale* **4**, 486-495 (2012).
19. Chow, E.K., *et al.* Nanodiamond Therapeutic Delivery Agents Mediate Enhanced Chemoresistant Tumor Treatment. *Sci Transl Med* **3**(2011).
20. Brunetti, V., *et al.* InP/ZnS as a safer alternative to CdSe/ZnS core/shell quantum dots: in vitro and in vivo toxicity assessment. *Nanoscale* **5**, 307-317 (2013).
21. Lynch, I. & Dawson, K.A. Protein-nanoparticle interactions. *Nano Today* **3**, 40-47 (2008).
22. Arbab, A.S., *et al.* A model of lysosomal metabolism of dextran coated superparamagnetic iron oxide (SPIO) nanoparticles: implications for cellular magnetic resonance imaging. *NMR in Biomedicine* **18**, 383-389 (2005).
23. Lévy, M., *et al.* Degradability of superparamagnetic nanoparticles in a model of intracellular environment: follow-up of magnetic, structural and chemical properties. *Nanotechnology* **21**, 395103 (2010).
24. Cho, E.C., Xie, J., Wurm, P.A. & Xia, Y. Understanding the role of surface charges in cellular adsorption versus internalization by selectively removing gold nanoparticles on the cell surface with a I2/KI etchant. *Nano Letters* **9**, 1080-1084 (2009).
25. Sabella, S., *et al.* Toxicity of citrate-capped AuNPs: an in vitro and in vivo assessment. *J Nanopart Res* **13**, 6821-6835 (2011).
26. Thaxton, C.S., Daniel, W.L., Giljohann, D.A., Thomas, A.D. & Mirkin, C.A. Templated Spherical High Density Lipoprotein Nanoparticles. *J Am Chem Soc* **131**, 1384-+ (2009).
27. Ashfaq, U.A., Javed, T., Rehman, S., Nawaz, Z. & Riazuddin, S. Lysosomotropic agents as HCV entry inhibitors. *Virol J* **8**, 163 (2011).
28. Flora, S.J.S. & Pachauri, V. Chelation in Metal Intoxication. *International Journal of Environmental Research and Public Health* **7**, 2745-2788 (2010).
29. Soenen, S.J.H., Himmelreich, U., Nuytten, N. & De Cuyper, M. Cytotoxic effects of iron oxide nanoparticles and implications for safety in cell labelling. *Biomaterials* **32**, 195-205 (2011).
30. Luthman, M. & Holmgren, A. Rat liver thioredoxin and thioredoxin reductase: purification and characterization. *Biochemistry* **21**, 6628-6633 (1982).
